# Nuclear effects on the transverse momentum spectra of charged particles in pPb collisions at $$\sqrt{s_{_\mathrm {NN}}} =5.02$$$$\,\text {TeV}$$

**DOI:** 10.1140/epjc/s10052-015-3435-4

**Published:** 2015-05-29

**Authors:** V. Khachatryan, A. M. Sirunyan, A. Tumasyan, W. Adam, T. Bergauer, M. Dragicevic, J. Erö, M. Friedl, R. Frühwirth, V. M. Ghete, C. Hartl, N. Hörmann, J. Hrubec, M. Jeitler, W. Kiesenhofer, V. Knünz, M. Krammer, I. Krätschmer, D. Liko, I. Mikulec, D. Rabady, B. Rahbaran, H. Rohringer, R. Schöfbeck, J. Strauss, W. Treberer-Treberspurg, W. Waltenberger, C.-E. Wulz, V. Mossolov, N. Shumeiko, J. Suarez Gonzalez, S. Alderweireldt, M. Bansal, S. Bansal, T. Cornelis, E. A. De Wolf, X. Janssen, A. Knutsson, J. Lauwers, S. Luyckx, S. Ochesanu, R. Rougny, M. Van De Klundert, H. Van Haevermaet, P. Van Mechelen, N. Van Remortel, A. Van Spilbeeck, F. Blekman, S. Blyweert, J. D’Hondt, N. Daci, N. Heracleous, J. Keaveney, S. Lowette, M. Maes, A. Olbrechts, Q. Python, D. Strom, S. Tavernier, W. Van Doninck, P. Van Mulders, G. P. Van Onsem, I. Villella, C. Caillol, B. Clerbaux, G. De Lentdecker, D. Dobur, L. Favart, A. P. R. Gay, A. Grebenyuk, A. Léonard, A. Mohammadi, L. Perniè, A. Randle-conde, T. Reis, T. Seva, L. Thomas, C. Vander Velde, P. Vanlaer, J. Wang, F. Zenoni, V. Adler, K. Beernaert, L. Benucci, A. Cimmino, S. Costantini, S. Crucy, S. Dildick, A. Fagot, G. Garcia, J. Mccartin, A. A. Ocampo Rios, D. Ryckbosch, S. Salva Diblen, M. Sigamani, N. Strobbe, F. Thyssen, M. Tytgat, E. Yazgan, N. Zaganidis, S. Basegmez, C. Beluffi, G. Bruno, R. Castello, A. Caudron, L. Ceard, G. G. Da Silveira, C. Delaere, T. du Pree, D. Favart, L. Forthomme, A. Giammanco, J. Hollar, A. Jafari, P. Jez, M. Komm, V. Lemaitre, C. Nuttens, D. Pagano, L. Perrini, A. Pin, K. Piotrzkowski, A. Popov, L. Quertenmont, M. Selvaggi, M. Vidal Marono, J. M. Vizan Garcia, N. Beliy, T. Caebergs, E. Daubie, G. H. Hammad, W. L. Aldá Júnior, G. A. Alves, L. Brito, M. Correa Martins Junior, T. Dos Reis Martins, C. Mora Herrera, M. E. Pol, P. Rebello Teles, W. Carvalho, J. Chinellato, A. Custódio, E. M. Da Costa, D. De Jesus Damiao, C. De Oliveira Martins, S. Fonseca De Souza, H. Malbouisson, D. Matos Figueiredo, L. Mundim, H. Nogima, W. L. Prado Da Silva, J. Santaolalla, A. Santoro, A. Sznajder, E. J. Tonelli Manganote, A. Vilela Pereira, C. A. Bernardes, S. Dogra, T. R. Fernandez Perez Tomei, E. M. Gregores, P. G. Mercadante, S. F. Novaes, Sandra S. Padula, A. Aleksandrov, V. Genchev, R. Hadjiiska, P. Iaydjiev, A. Marinov, S. Piperov, M. Rodozov, G. Sultanov, M. Vutova, A. Dimitrov, I. Glushkov, L. Litov, B. Pavlov, P. Petkov, J. G. Bian, G. M. Chen, H. S. Chen, M. Chen, T. Cheng, R. Du, C. H. Jiang, R. Plestina, F. Romeo, J. Tao, Z. Wang, C. Asawatangtrakuldee, Y. Ban, S. Liu, Y. Mao, S. J. Qian, D. Wang, W. Zou, C. Avila, A. Cabrera, L. F. Chaparro Sierra, C. Florez, J. P. Gomez, B. Gomez Moreno, J. C. Sanabria, N. Godinovic, D. Lelas, D. Polic, I. Puljak, Z. Antunovic, M. Kovac, V. Brigljevic, K. Kadija, J. Luetic, D. Mekterovic, L. Sudic, A. Attikis, G. Mavromanolakis, J. Mousa, C. Nicolaou, F. Ptochos, P. A. Razis, M. Bodlak, M. Finger, M. Finger, Y. Assran, A. Ellithi Kamel, M. A. Mahmoud, A. Radi, M. Kadastik, M. Murumaa, M. Raidal, A. Tiko, P. Eerola, G. Fedi, M. Voutilainen, J. Härkönen, V. Karimäki, R. Kinnunen, M. J. Kortelainen, T. Lampén, K. Lassila-Perini, S. Lehti, T. Lindén, P. Luukka, T. Mäenpää, T. Peltola, E. Tuominen, J. Tuominiemi, E. Tuovinen, L. Wendland, J. Talvitie, T. Tuuva, M. Besancon, F. Couderc, M. Dejardin, D. Denegri, B. Fabbro, J. L. Faure, C. Favaro, F. Ferri, S. Ganjour, A. Givernaud, P. Gras, G. Hamel de Monchenault, P. Jarry, E. Locci, J. Malcles, J. Rander, A. Rosowsky, M. Titov, S. Baffioni, F. Beaudette, P. Busson, C. Charlot, T. Dahms, M. Dalchenko, L. Dobrzynski, N. Filipovic, A. Florent, R. Granier de Cassagnac, L. Mastrolorenzo, P. Miné, C. Mironov, I. N. Naranjo, M. Nguyen, C. Ochando, P. Paganini, S. Regnard, R. Salerno, J. B. Sauvan, Y. Sirois, C. Veelken, Y. Yilmaz, A. Zabi, J.-L. Agram, J. Andrea, A. Aubin, D. Bloch, J.-M. Brom, E. C. Chabert, C. Collard, E. Conte, J.-C. Fontaine, D. Gelé, U. Goerlach, C. Goetzmann, A.-C. Le Bihan, K. Skovpen, P. Van Hove, S. Gadrat, S. Beauceron, N. Beaupere, G. Boudoul, E. Bouvier, S. Brochet, C. A. Carrillo Montoya, J. Chasserat, R. Chierici, D. Contardo, P. Depasse, H. El Mamouni, J. Fan, J. Fay, S. Gascon, M. Gouzevitch, B. Ille, T. Kurca, M. Lethuillier, L. Mirabito, S. Perries, J. D. Ruiz Alvarez, D. Sabes, L. Sgandurra, V. Sordini, M. Vander Donckt, P. Verdier, S. Viret, H. Xiao, Z. Tsamalaidze, C. Autermann, S. Beranek, M. Bontenackels, M. Edelhoff, L. Feld, A. Heister, O. Hindrichs, K. Klein, A. Ostapchuk, F. Raupach, J. Sammet, S. Schael, J. F. Schulte, H. Weber, B. Wittmer, V. Zhukov, M. Ata, M. Brodski, E. Dietz-Laursonn, D. Duchardt, M. Erdmann, R. Fischer, A. Güth, T. Hebbeker, C. Heidemann, K. Hoepfner, D. Klingebiel, S. Knutzen, P. Kreuzer, M. Merschmeyer, A. Meyer, P. Millet, M. Olschewski, K. Padeken, P. Papacz, H. Reithler, S. A. Schmitz, L. Sonnenschein, D. Teyssier, S. Thüer, M. Weber, V. Cherepanov, Y. Erdogan, G. Flügge, H. Geenen, M. Geisler, W. Haj Ahmad, F. Hoehle, B. Kargoll, T. Kress, Y. Kuessel, A. Künsken, J. Lingemann, A. Nowack, I. M. Nugent, L. Perchalla, O. Pooth, A. Stahl, M. Aldaya Martin, I. Asin, N. Bartosik, J. Behr, U. Behrens, A. J. Bell, A. Bethani, K. Borras, A. Burgmeier, A. Cakir, L. Calligaris, A. Campbell, S. Choudhury, F. Costanza, C. Diez Pardos, G. Dolinska, S. Dooling, T. Dorland, G. Eckerlin, D. Eckstein, T. Eichhorn, G. Flucke, J. Garay Garcia, A. Geiser, P. Gunnellini, J. Hauk, M. Hempel, H. Jung, A. Kalogeropoulos, M. Kasemann, P. Katsas, J. Kieseler, C. Kleinwort, I. Korol, D. Krücker, W. Lange, J. Leonard, K. Lipka, A. Lobanov, W. Lohmann, B. Lutz, R. Mankel, I. Marfin, I.-A. Melzer-Pellmann, A. B. Meyer, G. Mittag, J. Mnich, A. Mussgiller, S. Naumann-Emme, A. Nayak, E. Ntomari, H. Perrey, D. Pitzl, R. Placakyte, A. Raspereza, P. M. Ribeiro Cipriano, B. Roland, E. Ron, M. Ö. Sahin, J. Salfeld-Nebgen, P. Saxena, T. Schoerner-Sadenius, M. Schröder, C. Seitz, S. Spannagel, A. D. R. Vargas Trevino, R. Walsh, C. Wissing, V. Blobel, M. Centis Vignali, A. R. Draeger, J. Erfle, E. Garutti, K. Goebel, M. Görner, J. Haller, M. Hoffmann, R. S. Höing, A. Junkes, H. Kirschenmann, R. Klanner, R. Kogler, J. Lange, T. Lapsien, T. Lenz, I. Marchesini, J. Ott, T. Peiffer, A. Perieanu, N. Pietsch, J. Poehlsen, T. Poehlsen, D. Rathjens, C. Sander, H. Schettler, P. Schleper, E. Schlieckau, A. Schmidt, M. Seidel, V. Sola, H. Stadie, G. Steinbrück, D. Troendle, E. Usai, L. Vanelderen, A. Vanhoefer, C. Barth, C. Baus, J. Berger, C. Böser, E. Butz, T. Chwalek, W. De Boer, A. Descroix, A. Dierlamm, M. Feindt, F. Frensch, M. Giffels, A. Gilbert, F. Hartmann, T. Hauth, U. Husemann, I. Katkov, A. Kornmayer, E. Kuznetsova, P. Lobelle Pardo, M. U. Mozer, T. Müller, Th. Müller, A. Nürnberg, G. Quast, K. Rabbertz, S. Röcker, H. J. Simonis, F. M. Stober, R. Ulrich, J. Wagner-Kuhr, S. Wayand, T. Weiler, R. Wolf, G. Anagnostou, G. Daskalakis, T. Geralis, V. A. Giakoumopoulou, A. Kyriakis, D. Loukas, A. Markou, C. Markou, A. Psallidas, I. Topsis-Giotis, A. Agapitos, S. Kesisoglou, A. Panagiotou, N. Saoulidou, E. Stiliaris, X. Aslanoglou, I. Evangelou, G. Flouris, C. Foudas, P. Kokkas, N. Manthos, I. Papadopoulos, E. Paradas, J. Strologas, G. Bencze, C. Hajdu, P. Hidas, D. Horvath, F. Sikler, V. Veszpremi, G. Vesztergombi, A. J. Zsigmond, N. Beni, S. Czellar, J. Karancsi, J. Molnar, J. Palinkas, Z. Szillasi, P. Raics, Z. L. Trocsanyi, B. Ujvari, S. K. Swain, S. B. Beri, V. Bhatnagar, R. Gupta, U. Bhawandeep, A. K. Kalsi, M. Kaur, R. Kumar, M. Mittal, N. Nishu, J. B. Singh, Ashok Kumar, Arun Kumar, S. Ahuja, A. Bhardwaj, B. C. Choudhary, A. Kumar, S. Malhotra, M. Naimuddin, K. Ranjan, V. Sharma, S. Banerjee, S. Bhattacharya, K. Chatterjee, S. Dutta, B. Gomber, Sa. Jain, Sh. Jain, R. Khurana, A. Modak, S. Mukherjee, D. Roy, S. Sarkar, M. Sharan, A. Abdulsalam, D. Dutta, S. Kailas, V. Kumar, A. K. Mohanty, L. M. Pant, P. Shukla, A. Topkar, T. Aziz, S. Banerjee, S. Bhowmik, R. M. Chatterjee, R. K. Dewanjee, S. Dugad, S. Ganguly, S. Ghosh, M. Guchait, A. Gurtu, G. Kole, S. Kumar, M. Maity, G. Majumder, K. Mazumdar, G. B. Mohanty, B. Parida, K. Sudhakar, N. Wickramage, H. Bakhshiansohi, H. Behnamian, S. M. Etesami, A. Fahim, R. Goldouzian, M. Khakzad, M. Mohammadi Najafabadi, M. Naseri, S. Paktinat Mehdiabadi, F. Rezaei Hosseinabadi, B. Safarzadeh, M. Zeinali, M. Felcini, M. Grunewald, M. Abbrescia, C. Calabria, S. S. Chhibra, A. Colaleo, D. Creanza, N. De Filippis, M. De Palma, L. Fiore, G. Iaselli, G. Maggi, M. Maggi, S. My, S. Nuzzo, A. Pompili, G. Pugliese, R. Radogna, G. Selvaggi, A. Sharma, L. Silvestris, R. Venditti, G. Zito, P. Verwilligen, G. Abbiendi, A. C. Benvenuti, D. Bonacorsi, S. Braibant-Giacomelli, L. Brigliadori, R. Campanini, P. Capiluppi, A. Castro, F. R. Cavallo, G. Codispoti, M. Cuffiani, G. M. Dallavalle, F. Fabbri, A. Fanfani, D. Fasanella, P. Giacomelli, C. Grandi, L. Guiducci, S. Marcellini, G. Masetti, A. Montanari, F. L. Navarria, A. Perrotta, A. M. Rossi, F. Primavera, T. Rovelli, G. P. Siroli, N. Tosi, R. Travaglini, S. Albergo, G. Cappello, M. Chiorboli, S. Costa, F. Giordano, R. Potenza, A. Tricomi, C. Tuve, G. Barbagli, V. Ciulli, C. Civinini, R. D’Alessandro, E. Focardi, E. Gallo, S. Gonzi, V. Gori, P. Lenzi, M. Meschini, S. Paoletti, G. Sguazzoni, A. Tropiano, L. Benussi, S. Bianco, F. Fabbri, D. Piccolo, R. Ferretti, F. Ferro, M. Lo Vetere, E. Robutti, S. Tosi, M. E. Dinardo, S. Fiorendi, S. Gennai, R. Gerosa, A. Ghezzi, P. Govoni, M. T. Lucchini, S. Malvezzi, R. A. Manzoni, A. Martelli, B. Marzocchi, D. Menasce, L. Moroni, M. Paganoni, D. Pedrini, S. Ragazzi, N. Redaelli, T. Tabarelli de Fatis, S. Buontempo, N. Cavallo, S. Di Guida, F. Fabozzi, A. O. M. Iorio, L. Lista, S. Meola, M. Merola, P. Paolucci, P. Azzi, N. Bacchetta, D. Bisello, A. Branca, R. Carlin, P. Checchia, M. Dall’Osso, T. Dorigo, M. Galanti, U. Gasparini, P. Giubilato, F. Gonella, A. Gozzelino, K. Kanishchev, S. Lacaprara, M. Margoni, A. T. Meneguzzo, F. Montecassiano, J. Pazzini, N. Pozzobon, P. Ronchese, F. Simonetto, E. Torassa, M. Tosi, P. Zotto, A. Zucchetta, G. Zumerle, M. Gabusi, S. P. Ratti, V. Re, C. Riccardi, P. Salvini, P. Vitulo, M. Biasini, G. M. Bilei, D. Ciangottini, L. Fanò, P. Lariccia, G. Mantovani, M. Menichelli, A. Saha, A. Santocchia, A. Spiezia, K. Androsov, P. Azzurri, G. Bagliesi, J. Bernardini, T. Boccali, G. Broccolo, R. Castaldi, M. A. Ciocci, R. Dell’Orso, S. Donato, G. Fedi, F. Fiori, L. Foà, A. Giassi, M. T. Grippo, F. Ligabue, T. Lomtadze, L. Martini, A. Messineo, C. S. Moon, F. Palla, A. Rizzi, A. Savoy-Navarro, A. T. Serban, P. Spagnolo, P. Squillacioti, R. Tenchini, G. Tonelli, A. Venturi, P. G. Verdini, C. Vernieri, L. Barone, F. Cavallari, G. D’imperio, D. Del Re, M. Diemoz, C. Jorda, E. Longo, F. Margaroli, P. Meridiani, F. Micheli, S. Nourbakhsh, G. Organtini, R. Paramatti, S. Rahatlou, C. Rovelli, F. Santanastasio, L. Soffi, P. Traczyk, N. Amapane, R. Arcidiacono, S. Argiro, M. Arneodo, R. Bellan, C. Biino, N. Cartiglia, S. Casasso, M. Costa, A. Degano, N. Demaria, L. Finco, C. Mariotti, S. Maselli, E. Migliore, V. Monaco, M. Musich, M. M. Obertino, G. Ortona, L. Pacher, N. Pastrone, M. Pelliccioni, G. L. Pinna Angioni, A. Potenza, A. Romero, M. Ruspa, R. Sacchi, A. Solano, A. Staiano, U. Tamponi, S. Belforte, V. Candelise, M. Casarsa, F. Cossutti, G. Della Ricca, B. Gobbo, C. La Licata, M. Marone, A. Schizzi, T. Umer, A. Zanetti, S. Chang, T. A. Kropivnitskaya, S. K. Nam, D. H. Kim, G. N. Kim, M. S. Kim, M. S. Kim, D. J. Kong, S. Lee, Y. D. Oh, H. Park, A. Sakharov, D. C. Son, T. J. Kim, J. Y. Kim, S. Song, S. Choi, D. Gyun, B. Hong, M. Jo, H. Kim, Y. Kim, B. Lee, K. S. Lee, S. K. Park, Y. Roh, M. Choi, J. H. Kim, I. C. Park, G. Ryu, M. S. Ryu, Y. Choi, Y. K. Choi, J. Goh, D. Kim, E. Kwon, J. Lee, I. Yu, A. Juodagalvis, J. R. Komaragiri, M. A. B. Md Ali, E. Casimiro Linares, H. Castilla-Valdez, E. De La Cruz-Burelo, I. Heredia-de La Cruz, A. Hernandez-Almada, R. Lopez-Fernandez, A. Sanchez-Hernandez, S. Carrillo Moreno, F. Vazquez Valencia, I. Pedraza, H. A. Salazar Ibarguen, A. Morelos Pineda, D. Krofcheck, P. H. Butler, S. Reucroft, A. Ahmad, M. Ahmad, Q. Hassan, H. R. Hoorani, W. A. Khan, T. Khurshid, M. Shoaib, H. Bialkowska, M. Bluj, B. Boimska, T. Frueboes, M. Górski, M. Kazana, K. Nawrocki, K. Romanowska-Rybinska, M. Szleper, P. Zalewski, G. Brona, K. Bunkowski, M. Cwiok, W. Dominik, K. Doroba, A. Kalinowski, M. Konecki, J. Krolikowski, M. Misiura, M. Olszewski, W. Wolszczak, P. Bargassa, C. Beir ao Da Cruz E Silva, P. Faccioli, P. G. Ferreira Parracho, M. Gallinaro, L. Lloret Iglesias, F. Nguyen, J. Rodrigues Antunes, J. Seixas, J. Varela, P. Vischia, S. Afanasiev, P. Bunin, M. Gavrilenko, I. Golutvin, I. Gorbunov, A. Kamenev, V. Karjavin, V. Konoplyanikov, A. Lanev, A. Malakhov, V. Matveev, P. Moisenz, V. Palichik, V. Perelygin, S. Shmatov, N. Skatchkov, V. Smirnov, A. Zarubin, V. Golovtsov, Y. Ivanov, V. Kim, P. Levchenko, V. Murzin, V. Oreshkin, I. Smirnov, V. Sulimov, L. Uvarov, S. Vavilov, A. Vorobyev, An. Vorobyev, Yu. Andreev, A. Dermenev, S. Gninenko, N. Golubev, M. Kirsanov, N. Krasnikov, A. Pashenkov, D. Tlisov, A. Toropin, V. Epshteyn, V. Gavrilov, N. Lychkovskaya, V. Popov, I. Pozdnyakov, G. Safronov, S. Semenov, A. Spiridonov, V. Stolin, E. Vlasov, A. Zhokin, V. Andreev, M. Azarkin, I. Dremin, M. Kirakosyan, A. Leonidov, G. Mesyats, S. V. Rusakov, A. Vinogradov, A. Belyaev, E. Boos, A. Demiyanov, A. Ershov, A. Gribushin, O. Kodolova, V. Korotkikh, I. Lokhtin, S. Obraztsov, S. Petrushanko, V. Savrin, A. Snigirev, I. Vardanyan, I. Azhgirey, I. Bayshev, S. Bitioukov, V. Kachanov, A. Kalinin, D. Konstantinov, V. Krychkine, V. Petrov, R. Ryutin, A. Sobol, L. Tourtchanovitch, S. Troshin, N. Tyurin, A. Uzunian, A. Volkov, P. Adzic, M. Ekmedzic, J. Milosevic, V. Rekovic, J. Alcaraz Maestre, C. Battilana, E. Calvo, M. Cerrada, M. Chamizo Llatas, N. Colino, B. De La Cruz, A. Delgado Peris, D. Domínguez Vázquez, A. Escalante Del Valle, C. Fernandez Bedoya, J. P. Fernández Ramos, J. Flix, M. C. Fouz, P. Garcia-Abia, O. Gonzalez Lopez, S. Goy Lopez, J. M. Hernandez, M. I. Josa, E. Navarro De Martino, A. Pérez-Calero Yzquierdo, J. Puerta Pelayo, A. Quintario Olmeda, I. Redondo, L. Romero, M. S. Soares, C. Albajar, J. F. de Trocóniz, M. Missiroli, D. Moran, H. Brun, J. Cuevas, J. Fernandez Menendez, S. Folgueras, I. Gonzalez Caballero, J. A. Brochero Cifuentes, I. J. Cabrillo, A. Calderon, J. Duarte Campderros, M. Fernandez, G. Gomez, A. Graziano, A. Lopez Virto, J. Marco, R. Marco, C. Martinez Rivero, F. Matorras, F. J. Munoz Sanchez, J. Piedra Gomez, T. Rodrigo, A. Y. Rodríguez-Marrero, A. Ruiz-Jimeno, L. Scodellaro, I. Vila, R. Vilar Cortabitarte, D. Abbaneo, E. Auffray, G. Auzinger, M. Bachtis, P. Baillon, A. H. Ball, D. Barney, A. Benaglia, J. Bendavid, L. Benhabib, J. F. Benitez, C. Bernet, P. Bloch, A. Bocci, A. Bonato, O. Bondu, C. Botta, H. Breuker, T. Camporesi, G. Cerminara, S. Colafranceschi, M. D’Alfonso, D. d’Enterria, A. Dabrowski, A. David, F. De Guio, A. De Roeck, S. De Visscher, E. Di Marco, M. Dobson, M. Dordevic, B. Dorney, N. Dupont-Sagorin, A. Elliott-Peisert, G. Franzoni, W. Funk, D. Gigi, K. Gill, D. Giordano, M. Girone, F. Glege, R. Guida, S. Gundacker, M. Guthoff, J. Hammer, M. Hansen, P. Harris, J. Hegeman, V. Innocente, P. Janot, K. Kousouris, K. Krajczar, P. Lecoq, C. Lourenço, N. Magini, L. Malgeri, M. Mannelli, J. Marrouche, L. Masetti, F. Meijers, S. Mersi, E. Meschi, F. Moortgat, S. Morovic, M. Mulders, L. Orsini, L. Pape, E. Perez, L. Perrozzi, A. Petrilli, G. Petrucciani, A. Pfeiffer, M. Pimiä, D. Piparo, M. Plagge, A. Racz, G. Rolandi, M. Rovere, H. Sakulin, C. Schäfer, C. Schwick, A. Sharma, P. Siegrist, P. Silva, M. Simon, P. Sphicas, D. Spiga, J. Steggemann, B. Stieger, M. Stoye, Y. Takahashi, D. Treille, A. Tsirou, G. I. Veres, N. Wardle, H. K. Wöhri, H. Wollny, W. D. Zeuner, W. Bertl, K. Deiters, W. Erdmann, R. Horisberger, Q. Ingram, H. C. Kaestli, D. Kotlinski, U. Langenegger, D. Renker, T. Rohe, F. Bachmair, L. Bäni, L. Bianchini, M. A. Buchmann, B. Casal, N. Chanon, G. Dissertori, M. Dittmar, M. Donegà, M. Dünser, P. Eller, C. Grab, D. Hits, J. Hoss, W. Lustermann, B. Mangano, A. C. Marini, M. Marionneau, P. Martinez Ruiz del Arbol, M. Masciovecchio, D. Meister, N. Mohr, P. Musella, C. Nägeli, F. Nessi-Tedaldi, F. Pandolfi, F. Pauss, M. Peruzzi, M. Quittnat, L. Rebane, M. Rossini, A. Starodumov, M. Takahashi, K. Theofilatos, R. Wallny, H. A. Weber, C. Amsler, M. F. Canelli, V. Chiochia, A. De Cosa, A. Hinzmann, T. Hreus, B. Kilminster, C. Lange, B. Millan Mejias, J. Ngadiuba, D. Pinna, P. Robmann, F. J. Ronga, S. Taroni, M. Verzetti, Y. Yang, M. Cardaci, K. H. Chen, C. Ferro, C. M. Kuo, W. Lin, Y. J. Lu, R. Volpe, S. S. Yu, P. Chang, Y. H. Chang, Y. W. Chang, Y. Chao, K. F. Chen, P. H. Chen, C. Dietz, U. Grundler, W.-S. Hou, K. Y. Kao, Y. F. Liu, R.-S. Lu, D. Majumder, E. Petrakou, Y. M. Tzeng, R. Wilken, B. Asavapibhop, G. Singh, N. Srimanobhas, N. Suwonjandee, A. Adiguzel, M. N. Bakirci, S. Cerci, C. Dozen, I. Dumanoglu, E. Eskut, S. Girgis, G. Gokbulut, E. Gurpinar, I. Hos, E. E. Kangal, A. Kayis Topaksu, G. Onengut, K. Ozdemir, S. Ozturk, A. Polatoz, D. Sunar Cerci, B. Tali, H. Topakli, M. Vergili, I. V. Akin, B. Bilin, S. Bilmis, H. Gamsizkan, B. Isildak, G. Karapinar, K. Ocalan, S. Sekmen, U. E. Surat, M. Yalvac, M. Zeyrek, E. A. Albayrak, E. Gülmez, M. Kaya, O. Kaya, T. Yetkin, K. Cankocak, F. I. Vardarlı, L. Levchuk, P. Sorokin, J. J. Brooke, E. Clement, D. Cussans, H. Flacher, J. Goldstein, M. Grimes, G. P. Heath, H. F. Heath, J. Jacob, L. Kreczko, C. Lucas, Z. Meng, D. M. Newbold, S. Paramesvaran, A. Poll, T. Sakuma, S. Senkin, V. J. Smith, T. Williams, A. Belyaev, C. Brew, R. M. Brown, D. J. A. Cockerill, J. A. Coughlan, K. Harder, S. Harper, E. Olaiya, D. Petyt, C. H. Shepherd-Themistocleous, A. Thea, I. R. Tomalin, W. J. Womersley, S. D. Worm, M. Baber, R. Bainbridge, O. Buchmuller, D. Burton, D. Colling, N. Cripps, P. Dauncey, G. Davies, M. Della Negra, P. Dunne, W. Ferguson, J. Fulcher, D. Futyan, G. Hall, G. Iles, M. Jarvis, G. Karapostoli, M. Kenzie, R. Lane, R. Lucas, L. Lyons, A.-M. Magnan, S. Malik, B. Mathias, J. Nash, A. Nikitenko, J. Pela, M. Pesaresi, K. Petridis, D. M. Raymond, S. Rogerson, A. Rose, C. Seez, P. Sharp, A. Tapper, M. Vazquez Acosta, T. Virdee, S. C. Zenz, J. E. Cole, P. R. Hobson, A. Khan, P. Kyberd, D. Leggat, D. Leslie, I. D. Reid, P. Symonds, L. Teodorescu, M. Turner, J. Dittmann, K. Hatakeyama, A. Kasmi, H. Liu, T. Scarborough, O. Charaf, S. I. Cooper, C. Henderson, P. Rumerio, A. Avetisyan, T. Bose, C. Fantasia, P. Lawson, C. Richardson, J. Rohlf, J. St. John, L. Sulak, J. Alimena, E. Berry, S. Bhattacharya, G. Christopher, D. Cutts, Z. Demiragli, N. Dhingra, A. Ferapontov, A. Garabedian, U. Heintz, G. Kukartsev, E. Laird, G. Landsberg, M. Luk, M. Narain, M. Segala, T. Sinthuprasith, T. Speer, J. Swanson, R. Breedon, G. Breto, M. Calderon De La Barca Sanchez, S. Chauhan, M. Chertok, J. Conway, R. Conway, P. T. Cox, R. Erbacher, M. Gardner, W. Ko, R. Lander, M. Mulhearn, D. Pellett, J. Pilot, F. Ricci-Tam, S. Shalhout, J. Smith, M. Squires, D. Stolp, M. Tripathi, S. Wilbur, R. Yohay, R. Cousins, P. Everaerts, C. Farrell, J. Hauser, M. Ignatenko, G. Rakness, E. Takasugi, V. Valuev, M. Weber, K. Burt, R. Clare, J. Ellison, J. W. Gary, G. Hanson, J. Heilman, M. Ivova Rikova, P. Jandir, E. Kennedy, F. Lacroix, O. R. Long, A. Luthra, M. Malberti, M. Olmedo Negrete, A. Shrinivas, S. Sumowidagdo, S. Wimpenny, J. G. Branson, G. B. Cerati, S. Cittolin, R. T. D’Agnolo, A. Holzner, R. Kelley, D. Klein, J. Letts, I. Macneill, D. Olivito, S. Padhi, C. Palmer, M. Pieri, M. Sani, V. Sharma, S. Simon, M. Tadel, Y. Tu, A. Vartak, C. Welke, F. Würthwein, A. Yagil, D. Barge, J. Bradmiller-Feld, C. Campagnari, T. Danielson, A. Dishaw, V. Dutta, K. Flowers, M. Franco Sevilla, P. Geffert, C. George, F. Golf, L. Gouskos, J. Incandela, C. Justus, N. Mccoll, J. Richman, D. Stuart, W. To, C. West, J. Yoo, A. Apresyan, A. Bornheim, J. Bunn, Y. Chen, J. Duarte, A. Mott, H. B. Newman, C. Pena, M. Pierini, M. Spiropulu, J. R. Vlimant, R. Wilkinson, S. Xie, R. Y. Zhu, V. Azzolini, A. Calamba, B. Carlson, T. Ferguson, Y. Iiyama, M. Paulini, J. Russ, H. Vogel, I. Vorobiev, J. P. Cumalat, W. T. Ford, A. Gaz, M. Krohn, E. Luiggi Lopez, U. Nauenberg, J. G. Smith, K. Stenson, K. A. Ulmer, S. R. Wagner, J. Alexander, A. Chatterjee, J. Chaves, J. Chu, S. Dittmer, N. Eggert, N. Mirman, G. Nicolas Kaufman, J. R. Patterson, A. Ryd, E. Salvati, L. Skinnari, W. Sun, W. D. Teo, J. Thom, J. Thompson, J. Tucker, Y. Weng, L. Winstrom, P. Wittich, D. Winn, S. Abdullin, M. Albrow, J. Anderson, G. Apollinari, L. A. T. Bauerdick, A. Beretvas, J. Berryhill, P. C. Bhat, G. Bolla, K. Burkett, J. N. Butler, H. W. K. Cheung, F. Chlebana, S. Cihangir, V. D. Elvira, I. Fisk, J. Freeman, Y. Gao, E. Gottschalk, L. Gray, D. Green, S. Grünendahl, O. Gutsche, J. Hanlon, D. Hare, R. M. Harris, J. Hirschauer, B. Hooberman, S. Jindariani, M. Johnson, U. Joshi, K. Kaadze, B. Klima, B. Kreis, S. Kwan, J. Linacre, D. Lincoln, R. Lipton, T. Liu, J. Lykken, K. Maeshima, J. M. Marraffino, V. I. Martinez Outschoorn, S. Maruyama, D. Mason, P. McBride, P. Merkel, K. Mishra, S. Mrenna, S. Nahn, C. Newman-Holmes, V. O’Dell, O. Prokofyev, E. Sexton-Kennedy, S. Sharma, A. Soha, W. J. Spalding, L. Spiegel, L. Taylor, S. Tkaczyk, N. V. Tran, L. Uplegger, E. W. Vaandering, R. Vidal, A. Whitbeck, J. Whitmore, F. Yang, D. Acosta, P. Avery, P. Bortignon, D. Bourilkov, M. Carver, D. Curry, S. Das, M. De Gruttola, G. P. Di Giovanni, R. D. Field, M. Fisher, I. K. Furic, J. Hugon, J. Konigsberg, A. Korytov, T. Kypreos, J. F. Low, K. Matchev, H. Mei, P. Milenovic, G. Mitselmakher, L. Muniz, A. Rinkevicius, L. Shchutska, M. Snowball, D. Sperka, J. Yelton, M. Zakaria, S. Hewamanage, S. Linn, P. Markowitz, G. Martinez, J. L. Rodriguez, T. Adams, A. Askew, J. Bochenek, B. Diamond, J. Haas, S. Hagopian, V. Hagopian, K. F. Johnson, H. Prosper, V. Veeraraghavan, M. Weinberg, M. M. Baarmand, M. Hohlmann, H. Kalakhety, F. Yumiceva, M. R. Adams, L. Apanasevich, D. Berry, R. R. Betts, I. Bucinskaite, R. Cavanaugh, O. Evdokimov, L. Gauthier, C. E. Gerber, D. J. Hofman, P. Kurt, D. H. Moon, C. O’Brien, I. D. Sandoval Gonzalez, C. Silkworth, P. Turner, N. Varelas, B. Bilki, W. Clarida, K. Dilsiz, M. Haytmyradov, J.-P. Merlo, H. Mermerkaya, A. Mestvirishvili, A. Moeller, J. Nachtman, H. Ogul, Y. Onel, F. Ozok, A. Penzo, R. Rahmat, S. Sen, P. Tan, E. Tiras, J. Wetzel, K. Yi, B. A. Barnett, B. Blumenfeld, S. Bolognesi, D. Fehling, A. V. Gritsan, P. Maksimovic, C. Martin, M. Swartz, P. Baringer, A. Bean, G. Benelli, C. Bruner, R. P. Kenny, M. Malek, M. Murray, D. Noonan, S. Sanders, J. Sekaric, R. Stringer, Q. Wang, J. S. Wood, I. Chakaberia, A. Ivanov, S. Khalil, M. Makouski, Y. Maravin, L. K. Saini, N. Skhirtladze, I. Svintradze, J. Gronberg, D. Lange, F. Rebassoo, D. Wright, A. Baden, A. Belloni, B. Calvert, S. C. Eno, J. A. Gomez, N. J. Hadley, R. G. Kellogg, T. Kolberg, Y. Lu, A. C. Mignerey, K. Pedro, A. Skuja, M. B. Tonjes, S. C. Tonwar, A. Apyan, R. Barbieri, G. Bauer, W. Busza, I. A. Cali, M. Chan, L. Di Matteo, G. Gomez Ceballos, M. Goncharov, D. Gulhan, M. Klute, Y. S. Lai, Y.-J. Lee, A. Levin, P. D. Luckey, T. Ma, C. Paus, D. Ralph, C. Roland, G. Roland, G. S. F. Stephans, F. Stöckli, K. Sumorok, D. Velicanu, J. Veverka, B. Wyslouch, M. Yang, M. Zanetti, V. Zhukova, B. Dahmes, A. Gude, S. C. Kao, K. Klapoetke, Y. Kubota, J. Mans, N. Pastika, R. Rusack, A. Singovsky, N. Tambe, J. Turkewitz, J. G. Acosta, S. Oliveros, E. Avdeeva, K. Bloom, S. Bose, D. R. Claes, A. Dominguez, R. Gonzalez Suarez, J. Keller, D. Knowlton, I. Kravchenko, J. Lazo-Flores, F. Meier, F. Ratnikov, G. R. Snow, M. Zvada, J. Dolen, A. Godshalk, I. Iashvili, A. Kharchilava, A. Kumar, S. Rappoccio, G. Alverson, E. Barberis, D. Baumgartel, M. Chasco, A. Massironi, D. M. Morse, D. Nash, T. Orimoto, D. Trocino, R. J. Wang, D. Wood, J. Zhang, K. A. Hahn, A. Kubik, N. Mucia, N. Odell, B. Pollack, A. Pozdnyakov, M. Schmitt, S. Stoynev, K. Sung, M. Velasco, S. Won, A. Brinkerhoff, K. M. Chan, A. Drozdetskiy, M. Hildreth, C. Jessop, D. J. Karmgard, N. Kellams, K. Lannon, S. Lynch, N. Marinelli, Y. Musienko, T. Pearson, M. Planer, R. Ruchti, G. Smith, N. Valls, M. Wayne, M. Wolf, A. Woodard, L. Antonelli, J. Brinson, B. Bylsma, L. S. Durkin, S. Flowers, A. Hart, C. Hill, R. Hughes, K. Kotov, T. Y. Ling, W. Luo, D. Puigh, M. Rodenburg, B. L. Winer, H. Wolfe, H. W. Wulsin, O. Driga, P. Elmer, J. Hardenbrook, P. Hebda, A. Hunt, S. A. Koay, P. Lujan, D. Marlow, T. Medvedeva, M. Mooney, J. Olsen, P. Piroué, X. Quan, H. Saka, D. Stickland, C. Tully, J. S. Werner, A. Zuranski, E. Brownson, S. Malik, H. Mendez, J. E. Ramirez Vargas, V. E. Barnes, D. Benedetti, D. Bortoletto, M. De Mattia, L. Gutay, Z. Hu, M. K. Jha, M. Jones, K. Jung, M. Kress, N. Leonardo, D. H. Miller, N. Neumeister, B. C. Radburn-Smith, X. Shi, I. Shipsey, D. Silvers, A. Svyatkovskiy, F. Wang, W. Xie, L. Xu, J. Zablocki, N. Parashar, J. Stupak, A. Adair, B. Akgun, K. M. Ecklund, F. J. M. Geurts, W. Li, B. Michlin, B. P. Padley, R. Redjimi, J. Roberts, J. Zabel, B. Betchart, A. Bodek, R. Covarelli, P. de Barbaro, R. Demina, Y. Eshaq, T. Ferbel, A. Garcia-Bellido, P. Goldenzweig, J. Han, A. Harel, A. Khukhunaishvili, S. Korjenevski, G. Petrillo, D. Vishnevskiy, R. Ciesielski, L. Demortier, K. Goulianos, C. Mesropian, S. Arora, A. Barker, J. P. Chou, C. Contreras-Campana, E. Contreras-Campana, D. Duggan, D. Ferencek, Y. Gershtein, R. Gray, E. Halkiadakis, D. Hidas, S. Kaplan, A. Lath, S. Panwalkar, M. Park, R. Patel, S. Salur, S. Schnetzer, S. Somalwar, R. Stone, S. Thomas, P. Thomassen, M. Walker, K. Rose, S. Spanier, A. York, O. Bouhali, A. Castaneda Hernandez, R. Eusebi, W. Flanagan, J. Gilmore, T. Kamon, V. Khotilovich, V. Krutelyov, R. Montalvo, I. Osipenkov, Y. Pakhotin, A. Perloff, J. Roe, A. Rose, A. Safonov, I. Suarez, A. Tatarinov, N. Akchurin, C. Cowden, J. Damgov, C. Dragoiu, P. R. Dudero, J. Faulkner, K. Kovitanggoon, S. Kunori, S. W. Lee, T. Libeiro, I. Volobouev, E. Appelt, A. G. Delannoy, S. Greene, A. Gurrola, W. Johns, C. Maguire, Y. Mao, A. Melo, M. Sharma, P. Sheldon, B. Snook, S. Tuo, J. Velkovska, M. W. Arenton, S. Boutle, B. Cox, B. Francis, J. Goodell, R. Hirosky, A. Ledovskoy, H. Li, C. Lin, C. Neu, J. Wood, C. Clarke, R. Harr, P. E. Karchin, C. Kottachchi Kankanamge Don, P. Lamichhane, J. Sturdy, D. A. Belknap, D. Carlsmith, M. Cepeda, S. Dasu, L. Dodd, S. Duric, E. Friis, R. Hall-Wilton, M. Herndon, A. Hervé, P. Klabbers, A. Lanaro, C. Lazaridis, A. Levine, R. Loveless, A. Mohapatra, I. Ojalvo, T. Perry, G. A. Pierro, G. Polese, I. Ross, T. Sarangi, A. Savin, W. H. Smith, D. Taylor, C. Vuosalo, N. Woods, The CMS Collaboration

**Affiliations:** 1 Yerevan Physics Institute, Yerevan, Armenia; 2 Institut für Hochenergiephysik der OeAW, Wien, Austria; 3 National Centre for Particle and High Energy Physics, Minsk, Belarus; 4 Universiteit Antwerpen, Antwerpen, Belgium; 5 Vrije Universiteit Brussel, Brussels, Belgium; 6 Université Libre de Bruxelles, Bruxelles, Belgium; 7 Ghent University, Ghent, Belgium; 8 Université Catholique de Louvain, Louvain-la-Neuve, Belgium; 9 Université de Mons, Mons, Belgium; 10 Centro Brasileiro de Pesquisas Fisicas, Rio de Janeiro, Brazil; 11 Universidade do Estado do Rio de Janeiro, Rio de Janeiro, Brazil; 12 Universidade Estadual Paulista, Universidade Federal do ABC, São Paulo, Brazil; 13 Institute for Nuclear Research and Nuclear Energy, Sofia, Bulgaria; 14 University of Sofia, Sofia, Bulgaria; 15 Institute of High Energy Physics, Beijing, China; 16 State Key Laboratory of Nuclear Physics and Technology, Peking University, Beijing, China; 17 Universidad de Los Andes, Bogota, Colombia; 18 Faculty of Electrical Engineering, Mechanical Engineering and Naval Architecture, University of Split, Split, Croatia; 19 Faculty of Science, University of Split, Split, Croatia; 20 Institute Rudjer Boskovic, Zagreb, Croatia; 21 University of Cyprus, Nicosia, Cyprus; 22 Charles University, Prague, Czech Republic; 23 Academy of Scientific Research and Technology of the Arab Republic of Egypt, Egyptian Network of High Energy Physics, Cairo, Egypt; 24 National Institute of Chemical Physics and Biophysics, Tallinn, Estonia; 25 Department of Physics, University of Helsinki, Helsinki, Finland; 26 Helsinki Institute of Physics, Helsinki, Finland; 27 Lappeenranta University of Technology, Lappeenranta, Finland; 28 DSM/IRFU, CEA/Saclay, Gif-sur-Yvette, France; 29 Laboratoire Leprince-Ringuet, Ecole Polytechnique, IN2P3-CNRS, Palaiseau, France; 30 Institut Pluridisciplinaire Hubert Curien, Université de Strasbourg, Université de Haute Alsace Mulhouse, CNRS/IN2P3, Strasbourg, France; 31 Centre de Calcul de l’Institut National de Physique Nucleaire et de Physique des Particules, CNRS/IN2P3, Villeurbanne, France; 32 Institut de Physique Nucléaire de Lyon, Université de Lyon, Université Claude Bernard Lyon 1, CNRS-IN2P3, Villeurbanne, France; 33 Institute of High Energy Physics and Informatization, Tbilisi State University, Tbilisi, Georgia; 34 I. Physikalisches Institut, RWTH Aachen University, Aachen, Germany; 35 III. Physikalisches Institut A, RWTH Aachen University, Aachen, Germany; 36 III. Physikalisches Institut B, RWTH Aachen University, Aachen, Germany; 37 Deutsches Elektronen-Synchrotron, Hamburg, Germany; 38 University of Hamburg, Hamburg, Germany; 39 Institut für Experimentelle Kernphysik, Karlsruhe, Germany; 40 Institute of Nuclear and Particle Physics (INPP), NCSR Demokritos, Aghia Paraskevi, Greece; 41 University of Athens, Athens, Greece; 42 University of Ioánnina, Ioánnina, Greece; 43 Wigner Research Centre for Physics, Budapest, Hungary; 44 Institute of Nuclear Research ATOMKI, Debrecen, Hungary; 45 University of Debrecen, Debrecen, Hungary; 46 National Institute of Science Education and Research, Bhubaneswar, India; 47 Panjab University, Chandigarh, India; 48 University of Delhi, Delhi, India; 49 Saha Institute of Nuclear Physics, Kolkata, India; 50 Bhabha Atomic Research Centre, Mumbai, India; 51 Tata Institute of Fundamental Research, Mumbai, India; 52 Institute for Research in Fundamental Sciences (IPM), Tehran, Iran; 53 University College Dublin, Dublin, Ireland; 54 INFN Sezione di Bari, Università di Bari, Politecnico di Bari, Bari, Italy; 55 INFN Sezione di Bologna, Università di Bologna, Bologna, Italy; 56 INFN Sezione di Catania, Università di Catania, CSFNSM, Catania, Italy; 57 INFN Sezione di Firenze, Università di Firenze, Firenze, Italy; 58 INFN Laboratori Nazionali di Frascati, Frascati, Italy; 59 INFN Sezione di Genova, Università di Genova, Genoa, Italy; 60 INFN Sezione di Milano-Bicocca, Università di Milano-Bicocca, Milan, Italy; 61 INFN Sezione di Napoli, Università di Napoli ’Federico II’, Università della Basilicata (Potenza), Università G. Marconi (Roma), Naples, Italy; 62 INFN Sezione di Padova, Università di Padova, Università di Trento (Trento), Padua, Italy; 63 INFN Sezione di Pavia, Università di Pavia, Pavia, Italy; 64 INFN Sezione di Perugia, Università di Perugia, Perugia, Italy; 65 INFN Sezione di Pisa, Università di Pisa, Scuola Normale Superiore di Pisa, Pisa, Italy; 66 INFN Sezione di Roma, Università di Roma, Rome, Italy; 67 INFN Sezione di Torino, Università di Torino, Università del Piemonte Orientale (Novara), Turin, Italy; 68 INFN Sezione di Trieste, Università di Trieste, Trieste, Italy; 69 Kangwon National University, Chunchon, Korea; 70 Kyungpook National University, Taegu, Korea; 71 Chonbuk National University, Chonju, Korea; 72 Chonnam National University, Institute for Universe and Elementary Particles, Kwangju, Korea; 73 Korea University, Seoul, Korea; 74 Seoul National University, Seoul, Korea; 75 University of Seoul, Seoul, Korea; 76 Sungkyunkwan University, Suwon, Korea; 77 Vilnius University, Vilnius, Lithuania; 78 National Centre for Particle Physics, Universiti Malaya, Kuala Lumpur, Malaysia; 79 Centro de Investigacion y de Estudios Avanzados del IPN, Mexico City, Mexico; 80 Universidad Iberoamericana, Mexico City, Mexico; 81 Benemerita Universidad Autonoma de Puebla, Puebla, Mexico; 82 Universidad Autónoma de San Luis Potosí, San Luis Potosí, Mexico; 83 University of Auckland, Auckland, New Zealand; 84 University of Canterbury, Christchurch, New Zealand; 85 National Centre for Physics, Quaid-I-Azam University, Islamabad, Pakistan; 86 National Centre for Nuclear Research, Swierk, Poland; 87 Institute of Experimental Physics, Faculty of Physics, University of Warsaw, Warsaw, Poland; 88 Laboratório de Instrumentação e Física Experimental de Partículas, Lisbon, Portugal; 89 Joint Institute for Nuclear Research, Dubna, Russia; 90 Petersburg Nuclear Physics Institute, Gatchina, St. Petersburg, Russia; 91 Institute for Nuclear Research, Moscow, Russia; 92 Institute for Theoretical and Experimental Physics, Moscow, Russia; 93 P. N. Lebedev Physical Institute, Moscow, Russia; 94 Skobeltsyn Institute of Nuclear Physics, Lomonosov Moscow State University, Moscow, Russia; 95 State Research Center of Russian Federation, Institute for High Energy Physics, Protvino, Russia; 96 Faculty of Physics and Vinca Institute of Nuclear Sciences, University of Belgrade, Belgrade, Serbia; 97 Centro de Investigaciones Energéticas Medioambientales y Tecnológicas (CIEMAT), Madrid, Spain; 98 Universidad Autónoma de Madrid, Madrid, Spain; 99 Universidad de Oviedo, Oviedo, Spain; 100 Instituto de Física de Cantabria (IFCA), CSIC-Universidad de Cantabria, Santander, Spain; 101 CERN, European Organization for Nuclear Research, Geneva, Switzerland; 102 Paul Scherrer Institut, Villigen, Switzerland; 103 Institute for Particle Physics, ETH Zurich, Zurich, Switzerland; 104 Universität Zürich, Zurich, Switzerland; 105 National Central University, Chung-Li, Taiwan; 106 National Taiwan University (NTU), Taipei, Taiwan; 107 Department of Physics, Faculty of Science, Chulalongkorn University, Bangkok, Thailand; 108 Cukurova University, Adana, Turkey; 109 Physics Department, Middle East Technical University, Ankara, Turkey; 110 Bogazici University, Istanbul, Turkey; 111 Istanbul Technical University, Istanbul, Turkey; 112 National Scientific Center, Kharkov Institute of Physics and Technology, Kharkov, Ukraine; 113 University of Bristol, Bristol, UK; 114 Rutherford Appleton Laboratory, Didcot, UK; 115 Imperial College, London, UK; 116 Brunel University, Uxbridge, UK; 117 Baylor University, Waco, USA; 118 The University of Alabama, Tuscaloosa, USA; 119 Boston University, Boston, USA; 120 Brown University, Providence, USA; 121 University of California, Davis, USA; 122 University of California, Los Angeles, USA; 123 University of California, Riverside, Riverside, USA; 124 University of California, San Diego, La Jolla, USA; 125 University of California, Santa Barbara, Santa Barbara USA; 126 California Institute of Technology, Pasadena, USA; 127 Carnegie Mellon University, Pittsburgh, USA; 128 University of Colorado at Boulder, Boulder, USA; 129 Cornell University, Ithaca, USA; 130 Fairfield University, Fairfield, USA; 131 Fermi National Accelerator Laboratory, Batavia, USA; 132 University of Florida, Gainesville, USA; 133 Florida International University, Miami, USA; 134 Florida State University, Tallahassee, USA; 135 Florida Institute of Technology, Melbourne, USA; 136 University of Illinois at Chicago (UIC), Chicago, USA; 137 The University of Iowa, Iowa City, USA; 138 Johns Hopkins University, Baltimore, USA; 139 The University of Kansas, Lawrence, USA; 140 Kansas State University, Manhattan, USA; 141 Lawrence Livermore National Laboratory, Livermore, USA; 142 University of Maryland, College Park, USA; 143 Massachusetts Institute of Technology, Cambridge, USA; 144 University of Minnesota, Minneapolis, USA; 145 University of Mississippi, Oxford, USA; 146 University of Nebraska-Lincoln, Lincoln, USA; 147 State University of New York at Buffalo, Buffalo, USA; 148 Northeastern University, Boston, USA; 149 Northwestern University, Evanston, USA; 150 University of Notre Dame, Notre Dame, USA; 151 The Ohio State University, Columbus, USA; 152 Princeton University, Princeton, USA; 153 University of Puerto Rico, Mayaguez, USA; 154 Purdue University, West Lafayette, USA; 155 Purdue University Calumet, Hammond, USA; 156 Rice University, Houston, USA; 157 University of Rochester, Rochester, USA; 158 The Rockefeller University, New York, USA; 159 Rutgers, The State University of New Jersey, Piscataway, USA; 160 University of Tennessee, Knoxville, USA; 161 Texas A&M University, College Station, USA; 162 Texas Tech University, Lubbock, USA; 163 Vanderbilt University, Nashville, USA; 164 University of Virginia, Charlottesville, USA; 165 Wayne State University, Detroit, USA; 166 University of Wisconsin, Madison, USA

**Keywords:** CMS, Physics, Relativistic heavy-ion collisions

## Abstract

Transverse momentum spectra of charged particles are measured by the CMS experiment at the CERN LHC in pPb collisions at $\sqrt{s_{_\mathrm {NN}}} =5.02$ $\,\text {TeV}$, in the range $0.4 < p_{\mathrm {T}} < 120$ ${\,\text {GeV/}c}$ and pseudorapidity $|\eta _\textsc {cm} | < 1.8$ in the proton–nucleon center-of-mass frame. For $p_{\mathrm {T}} <10$ ${\,\text {GeV/}c}$, the charged-particle production is asymmetric about $\eta _\textsc {cm} = 0$, with smaller yield observed in the direction of the proton beam, qualitatively consistent with expectations from shadowing in nuclear parton distribution functions (nPDF). A pp reference spectrum at $\sqrt{s}=5.02$ $\,\text {TeV}$ is obtained by interpolation from previous measurements at higher and lower center-of-mass energies. The $p_{\mathrm {T}}$ distribution measured in pPb collisions shows an enhancement of charged particles with $p_{\mathrm {T}} >20$ ${\,\text {GeV/}c}$ compared to expectations from the pp reference. The enhancement is larger than predicted by perturbative quantum chromodynamics calculations that include antishadowing modifications of nPDFs.

## Introduction

The central goal of the heavy ion experimental program at ultra-relativistic energies is to create a system of deconfined quarks and gluons, known as a quark–gluon plasma (QGP), and to study its properties as it cools down and transitions into a hadron gas. A key tool in the studies of the QGP is the phenomenon of jet quenching [1], in which the partons produced in hard scatterings lose energy through gluon radiation and elastic scattering in the hot and dense partonic medium [2]. Since high transverse momentum quarks and gluons fragment into jets of hadrons, one of the observable consequences of parton energy loss is the suppression of the yield of high-$p_{\mathrm {T}}$ particles in comparison to their production in proton–proton (pp) collisions. This suppression, studied as a function of the $p_{\mathrm {T}}$ and pseudorapidity ($\eta$) of the produced particle, is usually quantified in terms of the nuclear modification factor, defined as

1 $$\begin{aligned} R_\mathrm {AB}(p_{\mathrm {T}},\eta ) = \frac{1}{\langle \mathrm {T}_\mathrm {AB} \rangle } \frac{\mathrm{d}^2 N^\mathrm {AB} / \mathrm{d}p_{\mathrm {T}} \, \mathrm{d}\eta }{\mathrm{d}^2 \sigma ^{\mathrm {p}\mathrm {p}} / \mathrm{d}p_{\mathrm {T}} \, \mathrm{d}\eta }, \end{aligned}$$

where $N^\mathrm {AB}$ is the particle yield in a collision between nuclear species A and B, $\sigma ^{\mathrm {p}\mathrm {p}}$ is the corresponding cross section in pp collisions, and $\langle \mathrm {T}_\mathrm {AB} \rangle$ is the average nuclear overlap function [3] in the AB collision (in the case of proton–nucleus collisions, the quantity $\langle \mathrm {T}_\mathrm {AB} \rangle = \langle \mathrm {T}_\mathrm {pA} \rangle$ is called average nuclear thickness function). If nuclear collisions behave as incoherent superpositions of nucleon–nucleon collisions, a ratio of unity is expected. Departures from unity are indicative of final-state effects such as parton energy loss, and/or initial-state effects such as modifications of the nuclear parton distribution functions (nPDF) [4]. The nPDFs are constrained by measurements in lepton–nucleus deep-inelastic scattering (DIS) and Drell–Yan (DY) production of dilepton pairs from $\mathrm{q} \overline{\mathrm{q}}$ annihilation in proton–nucleus collisions [5]. In the small parton fractional momentum regime ($x \lesssim 0.01$), the nPDFs are found to be suppressed relative to the proton PDFs, a phenomenon commonly referred to as “shadowing” [6]. At small $x$, where the parton distributions are described theoretically by non-linear evolution equations in $x$, gluon saturation is predicted by the color glass condensate models [7–9]. For the $x$ regime $0.02 \lesssim x \lesssim 0.2$, the nPDFs are enhanced (“antishadowing”) relative to the free-nucleon PDFs [5].

To gain access to the properties of the QGP produced in heavy ion collisions it is necessary to separate the effects directly related to the hot partonic medium from those that are not, referred to as “cold nuclear matter” effects. In particular, nPDF effects are expected to play an important role in the interpretation of nuclear modification factors at the CERN LHC. Unfortunately, the existing nuclear DIS and DY measurements constrain only poorly the gluon distributions over much of the kinematic range of interest. High-$p_{\mathrm {T}}$ hadron production in proton–nucleus (or deuteron–nucleus) collisions provides a valuable reference for nucleus–nucleus collisions, as it probes initial-state nPDF modifications over a wide kinematic range and is expected to be largely free from the final-state effects that accompany QGP production [10].

The measurements of the nuclear modification factors of neutral pions and charged hadrons in the most central gold–gold (AuAu) collisions at the relativistic heavy ion collider (RHIC) [11–14] revealed a large suppression at high $p_{\mathrm {T}}$, reaching an $R_\mathrm {AA}$ as low as 0.2. In contrast, no such suppression was found at mid-rapidity in deuteron–gold collisions at the same energy [15–18]. These findings established parton energy loss, rather than initial-state effects [19], as the mechanism responsible for the modifications observed in AuAu collisions.

At the LHC, the charged-particle suppression in lead–lead (PbPb) collisions persists at least up to a $p_{\mathrm {T}}$ of 100${\,\text {GeV/}c}$  [20, 21]. In proton–lead (pPb) collisions, the ALICE Collaboration reported no significant deviations from unity in the charged-particle $R_\mathrm {pPb}$ up to $p_{\mathrm {T}} \approx 50$ ${\,\text {GeV/}c}$  [22]. The analysis presented here used data from CMS to extend the measurement of the charged-particle $R_\mathrm {pPb}$ out to $p_{\mathrm {T}} \approx 120$ ${\,\text {GeV/}c}$, with the aim of evaluating initial-state effects over a kinematic range similar to that explored through measurements in PbPb collisions [20].

Proton–nucleus collisions have already been used to assess the impact of cold nuclear matter on jet production at the LHC. The transverse momentum balance, azimuthal angle correlations, and pseudorapidity distributions of dijets have been measured as a function of the event activity, and no significant indication of jet quenching was found [23]. When normalized to unity, the minimum-bias dijet pseudorapidity distributions are found to be consistent with next-to-leading-order (NLO) perturbative quantum chromodynamic (pQCD) calculations only if nPDF modifications are included [24]. Similarly, inclusive jet $R_\mathrm {pPb}$ measurements are also found to be consistent with NLO pQCD predictions that include nPDF modifications [25, 26]. The measurement of the charged-particle spectra presented in this paper provides a comparison to theory that is sensitive to smaller $x$ values than those accessible in the jet measurements. However, it should be noted that the charged-particle $R_\mathrm {pPb}$ is dependent upon non-perturbative hadronization effects, some of which, such as gluon fragmentation into charged hadrons, are poorly constrained at the LHC energies [27].

The $p_{\mathrm {T}}$ distributions of inclusive charged particles in pPb collisions at a nucleon–nucleon center-of-mass energy of 5.02$\,\text {TeV}$ are presented in the range of $0.4<p_{\mathrm {T}} <120$ ${\,\text {GeV/}c}$. The measurement is performed in several pseudorapidity intervals over $|\eta _\textsc {cm} |< 1.8$. Here $\eta _\textsc {cm}$ is the pseudorapidity in the proton–nucleon center-of-mass frame. The nuclear modification factor is studied at mid-rapidity, $|\eta _\textsc {cm} |< 1$, and the forward-backward asymmetry of the yields, $Y_\text {asym}$, defined as

2 $$\begin{aligned} Y_\text {asym}^{(a,b)}(p_{\mathrm {T}}) = \frac{\int _{-b}^{-a}\mathrm{d}\eta _\textsc {cm} \, \mathrm{d}^2 N_\text {ch}(p_{\mathrm {T}}) / \mathrm{d}\eta _\textsc {cm} \, \mathrm{d}p_{\mathrm {T}}}{\int _{a}^{b}\mathrm{d}\eta _\textsc {cm} \, \mathrm{d}^2 N_\text {ch}(p_{\mathrm {T}}) / \mathrm{d}\eta _\textsc {cm} \, \mathrm{d}p_{\mathrm {T}}}, \end{aligned}$$

is presented for three pseudorapidity intervals, where $a$ and $b$ are positive numbers, and $N_\text {ch}$ is the yield of charged particles.

Due to their wide kinematic coverage, the measurements are expected to provide information about the nPDFs in both the shadowing and antishadowing regions. In particular, the effects of shadowing are expected to be more prominent at forward pseudorapidities (in the proton-going direction), where smaller $x$ fractions in the nucleus are accessed.

In the absence of other competing effects, shadowing in the Pb nPDFs would result in values of $Y_\text {asym}>1$ at low $p_{\mathrm {T}}$ (i.e., small $x$). The effects of antishadowing can be probed with the $R_\mathrm {pPb}$ measurement at larger $p_{\mathrm {T}}$ values of $30 \lesssim p_{\mathrm {T}} \lesssim 100$ ${\,\text {GeV/}c}$ that correspond approximately to $0.02 \lesssim x \lesssim 0.2$. Antishadowing in the nPDFs may increase the yield of charged particles in pPb collisions compared with expectations from the yield in pp collisions.

## Data selection and analysis

### Experimental setup

A detailed description of the CMS detector can be found in Ref. [28]. The CMS experiment uses a right-handed coordinate system, with the origin at the nominal interaction point (IP) at the center of the detector, and the $z$ axis along the beam direction. The silicon tracker, located within the 3.8 $\text {\,T}$ magnetic field of the superconducting solenoid, is used to reconstruct charged-particle tracks. Consisting of 1440 silicon pixel detector modules and 15,148 silicon strip detector modules, totaling about 10 million silicon strips and 60 million pixels, the silicon tracker measures the tracks of charged particles within the pseudorapidity range $|\eta |< 2.5$. It provides an impact parameter resolution of $\approx 15\,\mu \text {m}$ and a $p_{\mathrm {T}}$ resolution of about 1.5 % for particles with $p_{\mathrm {T}}$ of 100${\,\text {GeV/}c}$. An electromagnetic calorimeter (ECAL) and a hadron calorimeter (HCAL) are also located inside the solenoid. The ECAL consists of more than 75, 000 lead tungstate crystals, arranged in a quasi-projective geometry; the crystals are distributed in a barrel region ($|\eta | < 1.48$) and in two endcaps that extend out to $|\eta | \approx 3.0$. The HCAL barrel and endcaps, hadron sampling calorimeters composed of brass and scintillator plates, have an acceptance of $|\eta | \lesssim 3.0$. The hadron forward calorimeters (HF), consisting of iron with quartz fibers read out by photomultipliers, extend the calorimeter coverage out to $|\eta | = 5.2$, and are used in offline event selection. Beam Pick-up Timing for the eXperiments (BPTX) devices were used to trigger the detector readout. They are located around the beam pipe at a distance of 175$\text {\,m}$ from the IP on either side, and are designed to provide precise information on the LHC bunch structure and timing of the incoming beams. The detailed Monte Carlo (MC) simulation of the CMS detector response is based on Geant4  [29].

This measurement is based on a data sample corresponding to an integrated luminosity of 35$\,\text {nb}^{-1}$, collected by the CMS experiment in pPb collisions during the 2013 LHC running period. The center-of-mass energy per nucleon pair was $\sqrt{s_{_\mathrm {NN}}} =5.02$ $\,\text {TeV}$, corresponding to per-nucleon beam energies of 4$\,\text {TeV}$ and 1.58$\,\text {TeV}$ for protons and lead nuclei, respectively. The data were taken with two beam configurations. Initially, the Pb nuclei traveled in the counterclockwise direction, while in the second data-taking period, the beam directions were reversed. Both data sets, the second one being larger by approximately 50 %, were analyzed independently, yielding compatible results. To combine data from the two beam configurations, results from the first data-taking period are reflected along the $z$-axis, so that in the combined analysis, the proton travels in the positive $z$ and $\eta$ directions. In this convention, massless particles emitted at $\eta _\textsc {cm} = 0$ in the nucleon–nucleon center-of-mass frame will be detected at $\eta _\text {lab} = 0.465$ in the laboratory frame. A symmetric region about $\eta _\textsc {cm} = 0$ is used in the data analysis, resulting in a selected pseudorapidity range of $|\eta _\textsc {cm} | < 1.8$.

### Event selection

The CMS online event selection employs a hardware-based level-1 (L1) trigger and a software-based high-level trigger (HLT). A minimum-bias sample is selected first by the L1 requirement of a pPb bunch crossing at the IP (as measured by the BPTX), and an HLT requirement of at least one reconstructed track with $p_{\mathrm {T}} >0.4$ ${\,\text {GeV/}c}$ in the pixel tracker. For most of the 5.02$\,\text {TeV}$ data collection, the minimum-bias trigger is significantly prescaled because of the high instantaneous LHC luminosity. To increase the $p_{\mathrm {T}}$ reach of the measurement, a set of more selective triggers is also used: additional L1 requirements are imposed to select events that have at least one reconstructed calorimeter jet with an uncorrected transverse energy of $E_{\mathrm {T}} > 12$ $\,\text {GeV}$, and $E_{\mathrm {T}} > 16$ $\,\text {GeV}$. These event selections are complemented by additional HLT requirements that select events based on the presence of at least one track with $p_{\mathrm {T}} > 12$ ${\,\text {GeV/}c}$ (for L1 $E_{\mathrm {T}} > 12\,\text {GeV}$), or with $p_{\mathrm {T}} > 20$ or 30${\,\text {GeV/}c}$ (for L1 $E_{\mathrm {T}} > 16\,\text {GeV}$) reconstructed in the pixel and strip tracker.

The above triggering strategy allows for the optimization of the data-taking rate while adequately sampling all $p_{\mathrm {T}}$ regions, including collecting all events containing very high-$p_{\mathrm {T}}$ tracks. The track trigger with a $p_{\mathrm {T}}$ threshold of 12${\,\text {GeV/}c}$ records about 140 times more events with high-$p_{\mathrm {T}}$ tracks than the minimum-bias trigger, the track $p_{\mathrm {T}} >20$ ${\,\text {GeV/}c}$ trigger enhances this with an additional factor of about 8, while the track $p_{\mathrm {T}} >30$ ${\,\text {GeV/}c}$ trigger that is not prescaled, increases the number of events with a high-$p_{\mathrm {T}}$ track by yet another factor of about 2.

In the offline analysis, additional requirements are applied. Events are accepted if they have (i) at least one HF calorimeter tower on both the positive and negative sides of the HF with more than $3\,\text {GeV}$ of total energy, (ii) at least one reconstructed collision vertex with two or more associated tracks, and (iii) a maximum distance of 15$\,\text {cm}$ along the beam axis between the vertex with the largest number of associated tracks and the nominal IP. Beam-related background is suppressed by rejecting events where less than 25 % of all reconstructed tracks are of good quality [30].

An event-by-event weight factor accounts for correcting the measured charged-particle spectra in pPb collisions to a detector-independent class of collisions termed as “double-sided” (DS) events, which are very similar to those that pass the offline selection described above. A DS event is defined as a collision producing at least one particle in the pseudorapidity range $-5<\eta _\text {lab}<-3$ and another in the range $3<\eta _\text {lab}<5$, each with proper lifetime $\tau > 10^{-18}$ $\text {\,s}$ and energy $E > 3$ $\,\text {GeV}$  [31]. The performance of the minimum-bias and high-$p_{\mathrm {T}}$ single-track triggers, as well as the offline criteria in selecting DS events, is evaluated with simulations using the hijing MC generator [32], version 1.383 [33], and the correction factors are computed as a function of the event multiplicity. An efficiency of 99 % is obtained for the minimum-bias trigger and a negligible correction (i.e., 100 % efficiency) for the high-$p_{\mathrm {T}}$ track-triggered events. The correction factor is also evaluated using an epos [34] simulation and, based on the difference between both generators, a slightly $p_{\mathrm {T}}$-dependent systematic uncertainty of 1 % is assigned to the final spectra.

During the pPb data taking period, about 3 % of the recorded events contained more than one pPb collision. To reduce potential bias in the measurements arising from such “pileup”, events with multiple reconstructed vertices are removed if the longitudinal distance between the vertices along the beamline is greater than a specific value that is related to the uncertainty of the vertex position. This value is also dependent on the number of tracks associated with each vertex and ranges from 0.2$\,\text {cm}$ for vertex pairs with at least 25 tracks associated with each vertex, to 3$\,\text {cm}$ for vertex pairs with only 3 tracks associated with the vertex having the fewest associated tracks. Simulated hijing events are used to tune the pileup-rejection algorithm in order to reduce the number of erroneously eliminated single-collision events to a negligible fraction, and still maintain a high rejection efficiency for genuine pileup events. The pileup-rejection efficiency is found to be $92\pm 2\,\%$, which is confirmed by using a low bunch intensity control sample in data.

**Fig. 1:**
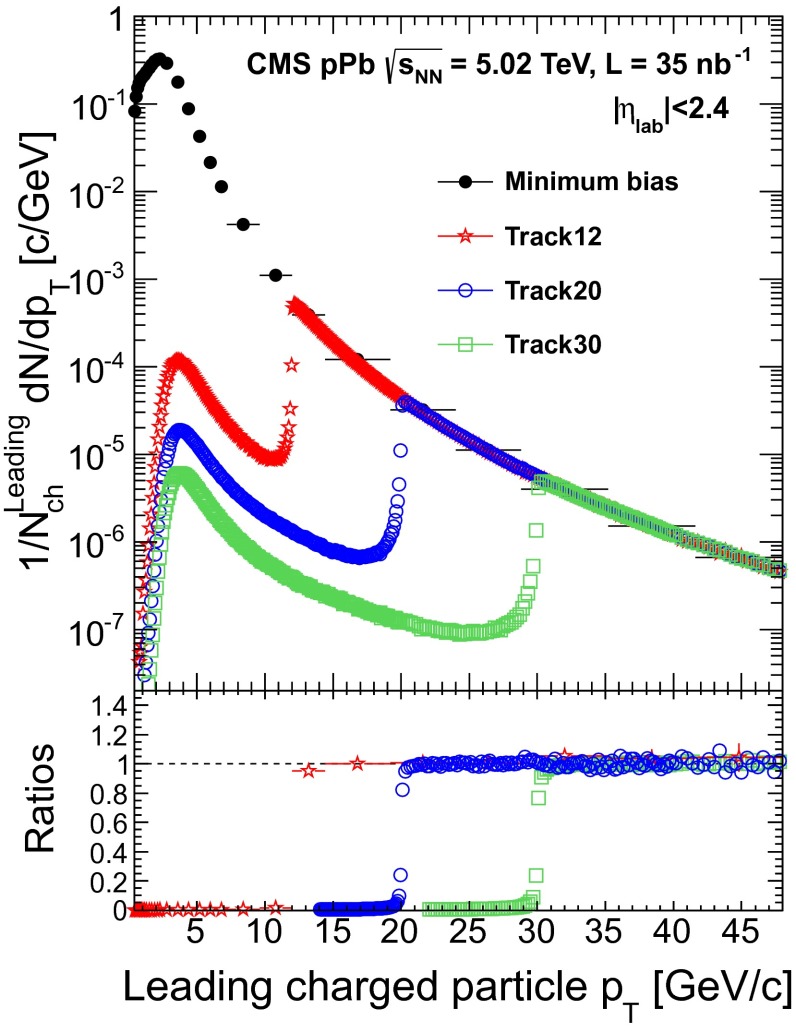
*Top* Charged-particle yields for the different triggers normalized to the number of leading charged particles with $p_{\mathrm {T}} >0.4$ ${\,\text {GeV/}c}$ in double-sided events, $N_\text {ch}^\text {Leading}$, as a function of leading-track $p_{\mathrm {T}}$. The track-triggered distributions are normalized by the number of leading tracks in regions not affected by the rapid rise of the trigger efficiency near threshold. *Bottom* Ratios of the leading-track $p_{\mathrm {T}}$ distributions for the four different triggers. The *stars* indicate the ratio of the 12${\,\text {GeV/}c}$ over the minimum-bias samples, the *circles* the 20 over the 12${\,\text {GeV/}c}$ samples, and the *squares* the ratio of the 30 over the 20${\,\text {GeV/}c}$ track-triggered spectra

To obtain inclusive particle spectra up to $p_{\mathrm {T}} \approx 120$ ${\,\text {GeV/}c}$, data recorded with the minimum-bias and high-$p_{\mathrm {T}}$ track triggers must be combined appropriately. The corresponding weight factors are computed by counting the number of events that contain leading tracks (defined as the track with the highest $p_{\mathrm {T}}$ in the event) in the range of $|\eta _\text {lab} |<2.4$ with $p_{\mathrm {T}}$ values in regions not affected by trigger thresholds, i.e., where the trigger efficiency of the higher-threshold trigger is constant relative to that of the lower-threshold trigger. The ratio of the number of such events in the two triggered sets of data are used as weight factors. For example, the region above which the track trigger with a $p_{\mathrm {T}}$ threshold of 12${\,\text {GeV/}c}$ has constant efficiency is determined by comparing the $p_{\mathrm {T}}$ distribution of the leading tracks to that of the minimum-bias data. Similarly, the constant efficiency region of the 20${\,\text {GeV/}c}$ track trigger is determined by comparison to the 12${\,\text {GeV/}c}$ track trigger, and the 30${\,\text {GeV/}c}$ trigger to the 20${\,\text {GeV/}c}$ trigger. The regions of constant efficiency for each trigger, as a function of leading charged-particle $p_{\mathrm {T}}$, are shown in Fig. 1. The 12, 20, and 30${\,\text {GeV/}c}$ triggers have constant efficiencies above a leading charged-particle $p_{\mathrm {T}}$ of 14, 22, and 32${\,\text {GeV/}c}$, respectively. The weight factors are then computed using the leading-track $p_{\mathrm {T}}$ classes of $14<p_{\mathrm {T}} <22$ ${\,\text {GeV/}c}$, $22<p_{\mathrm {T}} <32$ ${\,\text {GeV/}c}$, and $p_{\mathrm {T}} >32$ ${\,\text {GeV/}c}$ for the three high-$p_{\mathrm {T}}$ triggers. The combined uncertainty in these normalizations is dominated by the matching of the 12${\,\text {GeV/}c}$ track-triggered events to the minimum-bias events.

Some events selected by the track triggers in Fig. 1 are observed to result in a leading charged-particle $p_{\mathrm {T}}$ below the corresponding trigger threshold. This can happen if the $\eta$ of the track above threshold is outside the $\eta$ range considered in the analysis, and because the final track reconstruction—described in Sect. 2.3—is more robust and selective than the fast-tracking algorithm implemented in the HLT. When the HLT selects an event based on a misreconstructed track, it is often the case that the track is not found in the final reconstruction. To determine the inclusive particle spectrum, events are first uniquely classified into leading-track $p_{\mathrm {T}}$ classes in the pseudorapidity range in which the spectrum is being measured. The spectra are constructed by taking events from the minimum-bias, 12${\,\text {GeV/}c}$ track, 20${\,\text {GeV/}c}$ track, and 30${\,\text {GeV/}c}$ track trigger, respectively, for each bin. A 4 % systematic uncertainty on the possible trigger-bias effect is estimated from MC simulations. This procedure was verified in a data-driven way by constructing a charged-particle spectrum from an alternative combination of event samples triggered by reconstructed jets. Both final spectra, triggered by tracks and jets, are found to be consistent within the associated systematic uncertainty.

### Track reconstruction

The $p_{\mathrm {T}}$ distribution in this analysis corresponds to that of primary charged particles, defined as all charged particles with a mean proper lifetime greater than 1$\,\text {cm}$/$c$, including the products of strong and electromagnetic decays, but excluding particles originating from secondary interactions in the detector material. Weak-decay products are considered primary charged particles only if they are the daughters of a particle with a mean proper lifetime of less than 1$\,\text {cm}$/$c$, produced in the collision.

Charged particles are reconstructed using the standard CMS combinatorial track finder [35]. The proportion of misreconstructed tracks in the sample is reduced by applying an optimized set of standard tracking-quality selections, as described in Ref. [35]. A reconstructed track is considered as a primary charged-particle candidate if the statistical significance of the observed distance of closest approach between the track and the reconstructed collision vertex is less than three standard deviations, under the hypothesis that the track originated from this vertex. In case an event has multiple reconstructed collision vertices but is not rejected by the pileup veto, the track distance is evaluated relative to the best reconstructed collision vertex, defined as the one associated with the largest number of tracks, or the one with the lowest $\chi ^{2}$ if multiple vertices have the same number of associated tracks. To remove tracks with poor momentum reconstruction, the relative uncertainty of the momentum measurement $\sigma (p_{\mathrm {T}})/p_{\mathrm {T}}$ is required to be less than 10 %. Only tracks that fall in the kinematic range of $|\eta _\text {lab} |<2.4$ and $p_{\mathrm {T}} > 0.4$ ${\,\text {GeV/}c}$ are selected for analysis to ensure high tracking efficiency (70–90 %) and low misreconstruction rates ($<$2 %).

The yields of charged particles in each $p_{\mathrm {T}}$ and $\eta$ bin are weighted by a factor that accounts for the geometrical acceptance of the detector, the efficiency of the reconstruction algorithm, the fraction of tracks corresponding to a non-primary charged particle, the fraction of misreconstructed tracks that do not correspond to any charged particle, and the fraction of multiply-reconstructed tracks, which belong to the same charged particle.

The various correction terms are estimated using simulated minimum-bias pPb events from the hijing event generator. To reduce the statistical uncertainty in the correction factors at high $p_{\mathrm {T}}$, samples of hijing events are also mixed with pp dijet events from the pythia MC generator [36] (version 6.423, tune D6T with CTEQ6L1 PDF for 2.76$\,\text {TeV}$, tune Z2 for 7$\,\text {TeV}$  [37]).

The efficiency of the charged-particle reconstruction as well as the misreconstruction rates are also evaluated using pPb events simulated with epos. Differences between the two MC models are mostly dominated by the fraction of charged particles consisting of strange and multi-strange baryons that are too short-lived to be reconstructed unless they are produced at very high $p_{\mathrm {T}}$. Such differences in particle species composition, which are largest for particles with $3\lesssim p_{\mathrm {T}} \lesssim 14$ ${\,\text {GeV/}c}$, are propagated as a systematic uncertainty in the measured spectra. Below this $p_{\mathrm {T}}$ range, the strange baryons are only a small fraction of the inclusive charged particles in either model, and the difference in reconstruction efficiency between particle species has less impact at even larger $p_{\mathrm {T}}$, as high-$p_{\mathrm {T}}$ multi-strange baryons can be directly tracked with high efficiency. Additional checks were performed by changing cutoffs imposed during track selection and in the determination of the corresponding MC-based corrections. The corresponding variations in the corrected yields amount to 1.2–4.0 % depending on the $p_{\mathrm {T}}$ region under consideration, and are included in the systematic uncertainty.

Finite bin-widths and finite transverse momentum resolution can deform a steeply falling $p_{\mathrm {T}}$ spectrum. The data are corrected for the finite bin-width effect as they will be compared to a pp reference spectrum obtained by interpolation. The binning corrections are derived by fitting the measured distribution and using the resulting fit function as a probability distribution to generate entries in a histogram with the same $p_{\mathrm {T}}$ binning as used in the measurement. The correction factors are then obtained from the ratio of entries in the bins of the histogram to the fit function evaluated at the centers of the bins. This correction amounts to 0–12 %, depending on $p_{\mathrm {T}}$. A similar method is used to evaluate the “smearing” effect of the finite $p_{\mathrm {T}}$ resolution on the binned distributions. It is found that the momentum measurement, which has a resolution of $\sigma (p_{\mathrm {T}})/p_{\mathrm {T}} \approx 1.5\,\%$ near a $p_{\mathrm {T}}$ of 100${\,\text {GeV/}c}$, is sufficiently precise to only have a negligible effect on the measured spectra and therefore no correction factor is applied. To account for possible incorrect determination of the momentum resolution from the simulation, the effects were again evaluated after increasing the value of $\sigma (p_{\mathrm {T}})/p_{\mathrm {T}}$ by an additional 0.01, which produces a maximal distortion in the spectrum at a given $p_{\mathrm {T}}$ of less than 1 %.

### Proton–proton reference spectrum

The pPb collisions occur at a center-of-mass energy of 5.02 TeV per nucleon pair. At this collision energy, no proton–proton collisions have been provided by particle accelerators yet. The pp results closest in center-of-mass energy ($\sqrt{s}$) and with similar acceptance are those measured at 2.76 and 7$\,\text {TeV}$ by the CMS experiment [20, 38]. The determination of the nuclear modification factor $R_\mathrm {AB}$ resides in an interpolated reference spectrum to be constructed from data at higher and lower energies. We follow the direct interpolation method developed in Ref. [38] using measured $p_{\mathrm {T}}$ spectra from inelastic collisions with $|\eta |<1.0$ at $\sqrt{s}=0.63$, 1.8, and 1.96$\,\text {TeV}$ collision energies from CDF [39, 40], and 0.9, 2.76, and 7$\,\text {TeV}$ collision energies from CMS [20, 38]. This interpolation can be performed either as a function of $p_{\mathrm {T}}$ or as a function of $x_\mathrm {T}\equiv 2p_\mathrm {T}c/\sqrt{s}$.

**Fig. 2:**
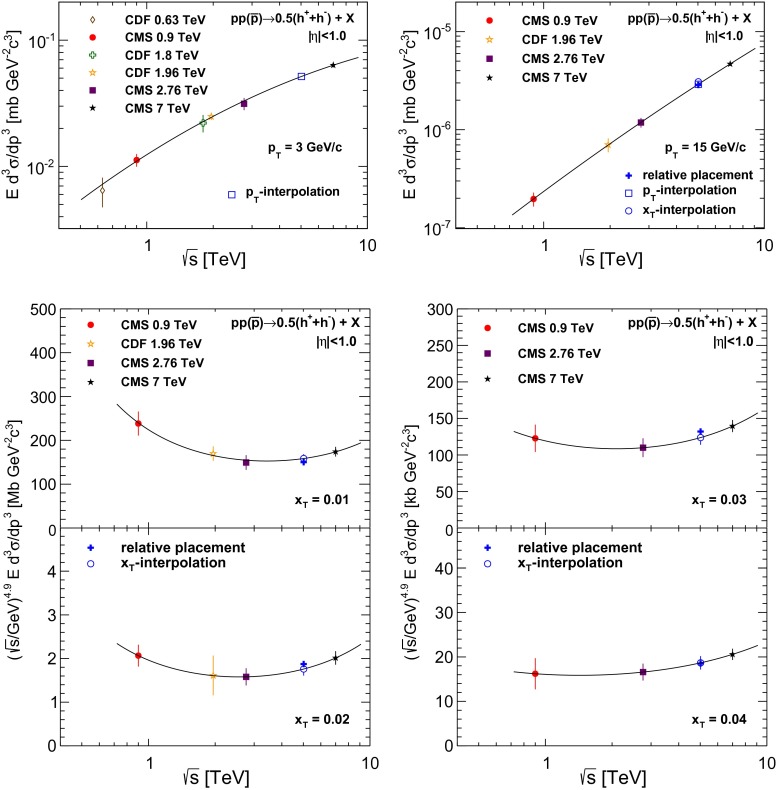
Examples of interpolations between measured charged-particle differential cross sections at different $\sqrt{s}$ for $p_{\mathrm {T}}$ values of 3 and 15${\,\text {GeV/}c}$ (*top left* and *right*), $x_\mathrm {T}$ values of 0.01 and 0.02 (*bottom left*), and $x_\mathrm {T}$ values of 0.03 and 0.04 (*bottom right*). These $x_\mathrm {T}$ values correspond to $p_{\mathrm {T}} \approx 25$, 50, 75, and 100${\,\text {GeV/}c}$ at $\sqrt{s}=5.02$ $\,\text {TeV}$. The second-order polynomial fits, performed in the plane of the log–log invariant production vs. $\sqrt{s}$, are shown by the *solid lines*. The *open squares* and *circles*, and the *filled crosses* represent interpolated cross section values at 5.02$\,\text {TeV}$ using different methods: $p_{\mathrm {T}}$-based interpolation, $x_\mathrm {T}$-based interpolation, and relative placement, respectively. The *error bars* on the interpolated points represent the uncertainties in the fit

Since the $p_{\mathrm {T}}$ or $x_\mathrm {T}$ values of the input data points are often different for each measurement performed at the various collision energies, each spectrum must first be fitted as a function of $p_{\mathrm {T}}$ or $x_\mathrm {T}$. An interpolation is performed by fitting each of the spectra to a power-law dependence, and the resulting residuals to first- or third-order splines. The fitted spectra are then interpolated to determine the value of the reference spectrum at $\sqrt{s}=5.02$ $\,\text {TeV}$ using a second-order polynomial in the plane of the log-log invariant production *vs.* $\sqrt{s}$, as shown in Fig. 2. For the $p_{\mathrm {T}}$-based direct interpolation, data from only two of the six spectra are available at $p_{\mathrm {T}} > 30$ ${\,\text {GeV/}c}$, which implies that the $p_{\mathrm {T}}$-based direct interpolation is well constrained only at low $p_{\mathrm {T}}$. On the other hand, the $x_\mathrm {T}$-based interpolation is well constrained at high $p_{\mathrm {T}}$ for $\sqrt{s}=5.02\,\text {TeV}$, so it is natural to combine the reference distributions from these two direct interpolation methods.

The final pp reference spectrum is obtained by combining the $p_{\mathrm {T}}$- and $x_\mathrm {T}$-based reference spectra as follows. The $p_{\mathrm {T}}$-based reference is chosen for $p_{\mathrm {T}}$ below 12.5${\,\text {GeV/}c}$, and the $x_\mathrm {T}$-based result above 13.5${\,\text {GeV/}c}$; between these two $p_{\mathrm {T}}$ values a linear weighting is implemented for the two references. The systematic uncertainty in the pp reference spectrum is determined through changing both the specific method of interpolation, as well as the underlying pp reference data within their statistical and systematic uncertainties. The systematic uncertainty is dominated by the interpolation method, and is determined by comparing the combined $p_{\mathrm {T}}$- and $x_\mathrm {T}$-based reference spectra to the reference spectra obtained solely from the $p_{\mathrm {T}}$ or $x_\mathrm {T}$ distributions, and also from a reference spectrum determined by a “relative placement” method. In the latter, the reference spectrum is obtained by computing where the 5.02$\,\text {TeV}$ spectrum is situated with respect to the 2.76 and 7$\,\text {TeV}$ spectra in pythia, and applying the computed placement factors to the measured 2.76 and 7$\,\text {TeV}$ spectra. The placement factors are determined by taking the value of the 5.02$\,\text {TeV}$ pythia spectrum, subtracting the value of the 2.76$\,\text {TeV}$ spectrum, and dividing by the difference between the 7 and the 2.76$\,\text {TeV}$ spectra. This process is then reversed by using the computed placement factors from pythia, and replacing the 2.76 and 7$\,\text {TeV}$ pythia spectra with the measured ones to determine the interpolated 5.02$\,\text {TeV}$ spectrum. Additionally, the NLO-based center-of-mass energy rescaling proposed in Ref. [41] is found to yield results consistent within the uncertainties of the other employed methods. The uncertainty in the pp reference distribution due to the interpolation method is estimated to amount to 10 %, which captures the overall point-to-point variations in all of the interpolation and scaling methods employed. The contribution from the uncertainties in the underlying pp input data corresponds to 6 %. These numbers are added in quadrature, resulting in the 12 % uncertainty quoted for the $\sqrt{s}=5.02$ $\,\text {TeV}$ interpolated pp reference spectrum.

## Systematic uncertainties

A summary of all the contributions to systematic uncertainties in the $p_{\mathrm {T}}$ spectra, $R^{*}_\mathrm {pPb}$, and $Y_\text {asym}$ are given in Table 1. The asterisk symbol is introduced to denote that an interpolated, rather than measured, pp reference spectrum is used to construct the nuclear modification factor. Aside from the uncertainty from the spectra relative normalization and average nuclear thickness, all uncertainties are determined by taking the approximate maximum deviation from the central value found for the given source. For the particle species uncertainty, an asymmetric uncertainty band is quoted because the maximum deviation above the central value of the measurement is much larger than the maximum deviation below. For the purpose of determining the significance of observed features in the measurement, the uncertainties are conservatively treated as following a Gaussian distribution with a root mean square given by the value of the uncertainty as determined above.

The degree of correlation among different uncertainties is described next. For the spectra and $R^{*}_\mathrm {pPb}$ measurements, the uncertainty in the efficiency of the single-track trigger and offline requirements in selecting DS events is largely a normalization uncertainty, although it also slightly affects the shape of the spectrum for $p_{\mathrm {T}} \lesssim 3 {\,\text {GeV/}c}$. The uncertainty from the contribution of the various particle species to the unidentified spectrum has the most significant effect in the region $3 < p_{\mathrm {T}} < 14 {\,\text {GeV/}c}$ and can impact the shape of the spectrum in a smooth fashion. At high $p_{\mathrm {T}}$, this effect is less prominent because, due to time dilation, unstable particles have a higher probability of traversing the inner tracker before decaying and therefore a higher probability of being reconstructed. Therefore, from this uncertainty the lower bound on the pPb spectra measurement at higher $p_{\mathrm {T}}$ is 2.5 % below the central value, which corresponds to no unstable particles being produced. Uncertainty in track misreconstruction can also affect the shape of the measured spectrum, as the misreconstructed fraction of high-$p_{\mathrm {T}}$ particles is sensitive to large occupancy in the silicon tracker within the cones of high-energy jets. The uncertainty in tracking selection can also affect the shape of the spectrum by raising or lowering the measured values at high $p_{\mathrm {T}}$, without changing the low-$p_{\mathrm {T}}$ values, as high-$p_{\mathrm {T}}$ tracks are more sensitive to possible mismodeling of detector alignment than low-$p_{\mathrm {T}}$ tracks. The uncertainty in the relative normalization of spectra is computed from the normalization factors involved in the combination of the $p_{\mathrm {T}}$ distributions from different triggers. This uncertainty only applies for selected $p_{\mathrm {T}}$ regions, and may raise or lower the spectrum above $p_{\mathrm {T}} =14$ ${\,\text {GeV/}c}$ by a constant factor of 1 % relative to the lower-$p_{\mathrm {T}}$ part of the spectrum. The uncertainty from potential biases of the method used to combine triggers can also affect the shape of the spectrum above $p_{\mathrm {T}}$ = 14${\,\text {GeV/}c}$.

For the $R^{*}_\mathrm {pPb}$ measurement, the uncertainty in the average nuclear thickness function [3] can influence the $R^{*}_\mathrm {pPb}$ curve by a constant multiplicative factor. The uncertainty from the pp interpolation is strongly correlated among points close together in $p_{\mathrm {T}}$, while some partial correlation remains throughout the whole $p_{\mathrm {T}}$ region, even for very different $p_{\mathrm {T}}$ values.

For the forward-backward asymmetry measurements, most of these uncertainties cancel in part or in full when the ratio of the spectra is taken. The remaining uncertainty in the detector acceptance and tracking efficiency can change the shape of the forward-backward asymmetry, particularly at high $p_{\mathrm {T}}$.

**Table 1:** Systematic uncertainties in the measurement of charged-particle spectra, $R^{*}_\mathrm {pPb}$, and $Y_\text {asym}$

Source	Uncertainty (%)
Trigger efficiency	1.0
Momentum resolution	1.0
Particle species composition	1–10.0 (0.5–5)
Track misreconstruction rate	1.0
Track selection	1.2–4.0
Spectra relative normalization	0.0–1.0
Trigger bias	0.0–4.0
Total (spectra)	2.2–10.9
pp interpolation	12.0
Total ($R^{*}_\mathrm {pPb}$)	12.2–16.2
$\langle \mathrm {T}_\mathrm {pPb} \rangle$ average nuclear thickness	4.8
Total ($Y_\text {asym}\quad 0.3< |\eta _\textsc {cm} | < 0.8$)	2.0–3.0
Total ($Y_\text {asym} \quad 0.8 < |\eta _\textsc {cm} | < 1.3$)	2.0–5.0
Total ($Y_\text {asym} \quad 1.3 < |\eta _\textsc {cm} | < 1.8$)	2.0–5.0

**Fig. 3:**
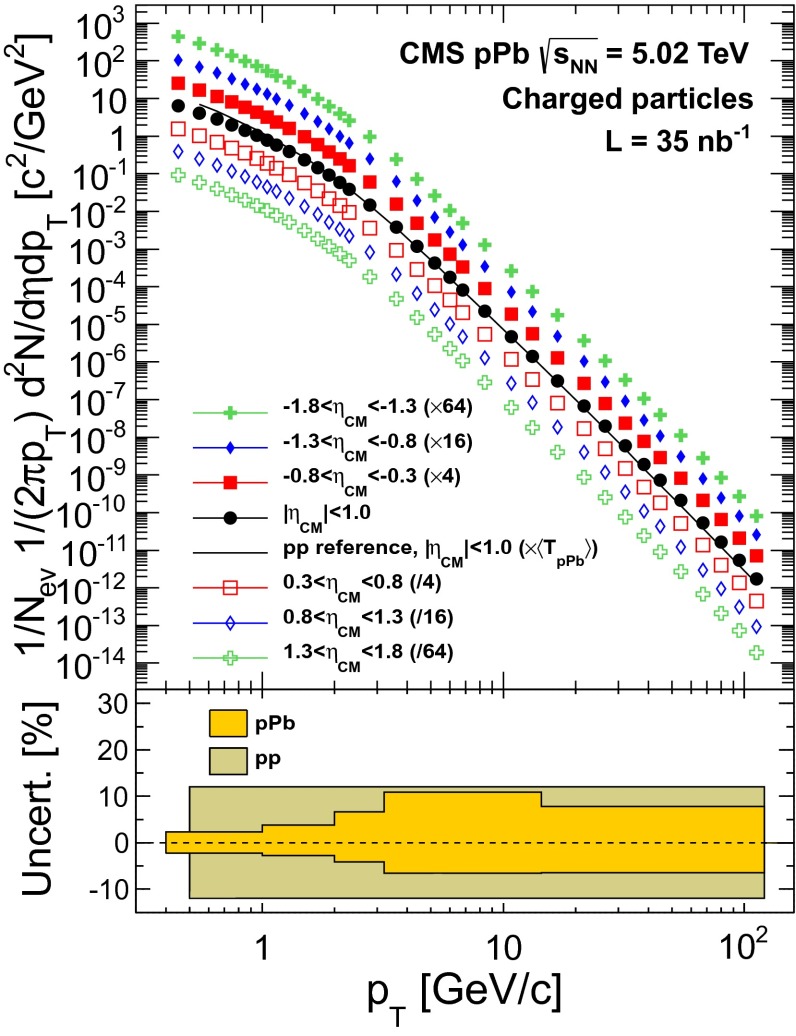
*Top* Measured charged-particle transverse momentum spectra in pPb collisions at $\sqrt{s_{_\mathrm {NN}}} =5.02$ $\,\text {TeV}$ for: $|\eta _\textsc {cm} |<1.0$, $0.3<\pm \eta _\textsc {cm} <0.8$, $0.8<\pm \eta _\textsc {cm} <1.3$, and $1.3<\pm \eta _\textsc {cm} <1.8$, and the interpolated pp reference spectrum in $|\eta _\textsc {cm} |<1$, normalized to the number of double-sided events. Positive pseudorapidity values correspond to the proton beam direction. The spectra have been scaled by the quoted factors to provide better visibility. *Bottom* Systematic uncertainties in the measured pPb and interpolated pp spectra, as a function of $p_{\mathrm {T}}$ (see text)

The ranges quoted refer to the variations of the uncertainties as a function of $p_{\mathrm {T}}$. Values in parentheses denote the negative part of the asymmetric uncertainty where applicable. The total uncertainties of the measured pPb and the interpolated pp spectra, as a function of $p_{\mathrm {T}}$, are shown in the lower panel of Fig. 3

## Results

The measured charged-particle yields in double-sided pPb collisions at $\sqrt{s_{_\mathrm {NN}}} =5.02\,\text {TeV}$ are plotted in Fig. 3 for the $|\eta _\textsc {cm} |<1.0$, $0.3<\pm \eta _\textsc {cm} <0.8$, $0.8<\pm \eta _\textsc {cm} <1.3$, and $1.3<\pm \eta _\textsc {cm} <1.8$ pseudorapidity ranges. Positive (negative) pseudorapidity values correspond to the proton (lead) beam direction. To improve the visibility of the results, the spectra at different pseudorapidities have been scaled up and down by multiple factors of 4 relative to the data for $|\eta _\mathrm{CM}|<1$. The relative uncertainties for the pPb and the pp spectra are given in the bottom panel.

The measurement of the charged-particle nuclear modification factor of Eq. (1) requires a rescaling of the pp cross section by the average nuclear thickness function in minimum-bias pPb collisions. This factor amounts to $\langle \mathrm {T}_\mathrm {pPb}\rangle =(0.0983\pm 0.0044)\,\mathrm {mb}^{-1}$ for inelastic pPb collisions and is obtained from a Glauber MC simulation [3, 42], where the Pb nucleus is described using a Woods-Saxon distribution with nuclear radius $6.62\pm 0.13$ $\text {\,fm}$ and skin depth of $0.546\pm 0.055$ $\text {\,fm}$  [3, 43]. As double-sided events correspond to 94–97 % of inelastic collisions based on HIJING and EPOS MC computations [31], the value of $\langle \mathrm {T}_\mathrm {pPb} \rangle$ would be about 5 % higher for double-sided events.

**Fig. 4:**
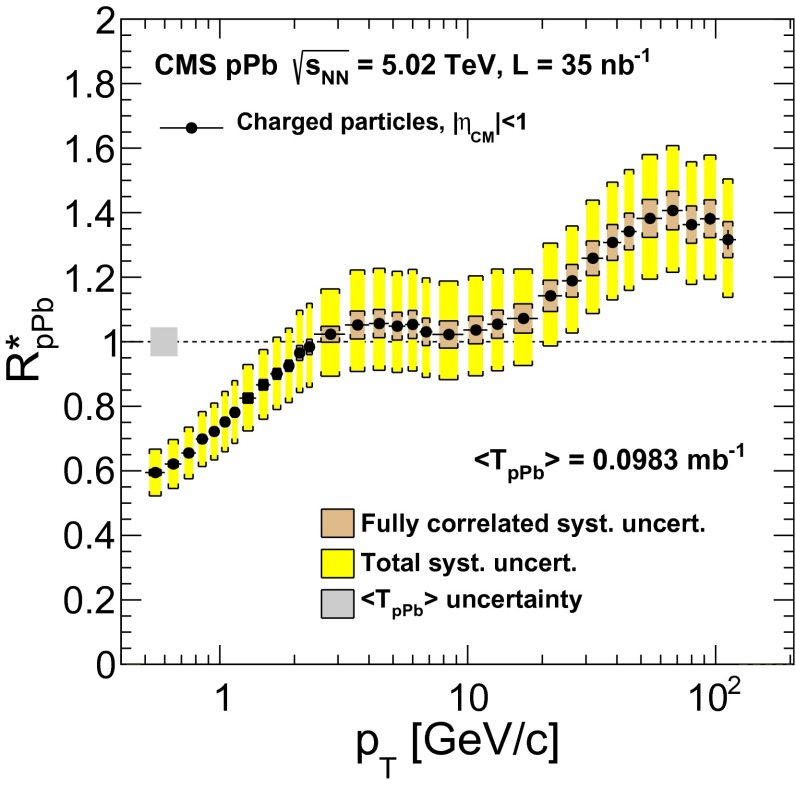
Measured nuclear modification factor as a function of $p_{\mathrm {T}}$ for charged particles produced in $|\eta _\textsc {cm} |<1$. The *shaded band* at unity and $p_{\mathrm {T}} \approx 0.6$ represents the uncertainty in the Glauber calculation of $\langle \mathrm {T}_\mathrm {pPb} \rangle$. The *smaller uncertainty band* around the data points shows the uncertainty from effects (combining spectra, track selection, and trigger efficiency) that are fully correlated in specific $p_{\mathrm {T}}$ regions. The total systematic uncertainties, dominated by uncertainty in the pp interpolation, are shown by the *larger band* (see Table 1)

The charged-particle $R^{*}_\mathrm {pPb}$ at mid-rapidity ($|\eta _\textsc {cm} |<1$) is plotted in Fig. 4 as a function of $p_{\mathrm {T}}$. The shaded band at unity and $p_{\mathrm {T}} \approx 0.6$ represents the uncertainty in the Glauber calculation of $\langle \mathrm {T}_\mathrm {pPb}\rangle$. The smaller uncertainty band around the measured values shows the fully correlated uncertainties from the following sources: spectra relative normalization, track selection, and trigger efficiency. The total systematic uncertainties are shown by the larger band around the measured values (Table 1). The nuclear modification factor shows a steady rise to unity at $p_{\mathrm {T}} \approx 2{\,\text {GeV/}c}$, then remains constant at unity up to approximately 20${\,\text {GeV/}c}$, and rises again at higher $p_{\mathrm {T}}$, reaching a maximum value around 1.3–1.4 above 40${\,\text {GeV/}c}$.

The fact that the nuclear modification factor is below unity for $p_{\mathrm {T}} \lesssim 2$ ${\,\text {GeV/}c}$ is anticipated since particle production in this region is dominated by softer scattering processes, that are not expected to scale with the nuclear thickness function. In the intermediate $p_{\mathrm {T}}$ range (2–5${\,\text {GeV/}c}$), no significant deviation from unity is found in the $R^{*}_\mathrm {pPb}$ ratio. There are several prior measurements that suggest an interplay of multiple effects in this $p_{\mathrm {T}}$ region. At lower collision energies, an enhancement (“Cronin effect” [44]) has been observed [15–18] that is larger for baryons than for mesons, and is stronger in the more central collisions. This enhancement has been attributed to a combination of initial-state multiple scattering effects, causing momentum broadening, and hadronization through parton recombination (a final-state effect) [45] invoked to accommodate baryon/meson differences. Recent results from pPb collisions at $\sqrt{s_{_\mathrm {NN}}} =5.02\,\text {TeV}$ [31, 46–49] and from dAu collisions at $\sqrt{s_{_\mathrm {NN}}}$ = 200$\,\text {GeV}$  [50, 51] suggest that collective effects may also play a role in the intermediate-$p_{\mathrm {T}}$ region. Most theoretical models do not predict a Cronin enhancement in this $p_{\mathrm {T}}$ range at LHC energies as the effect of initial-state multiple scattering is compensated by nPDF shadowing [52].

**Fig. 5:**
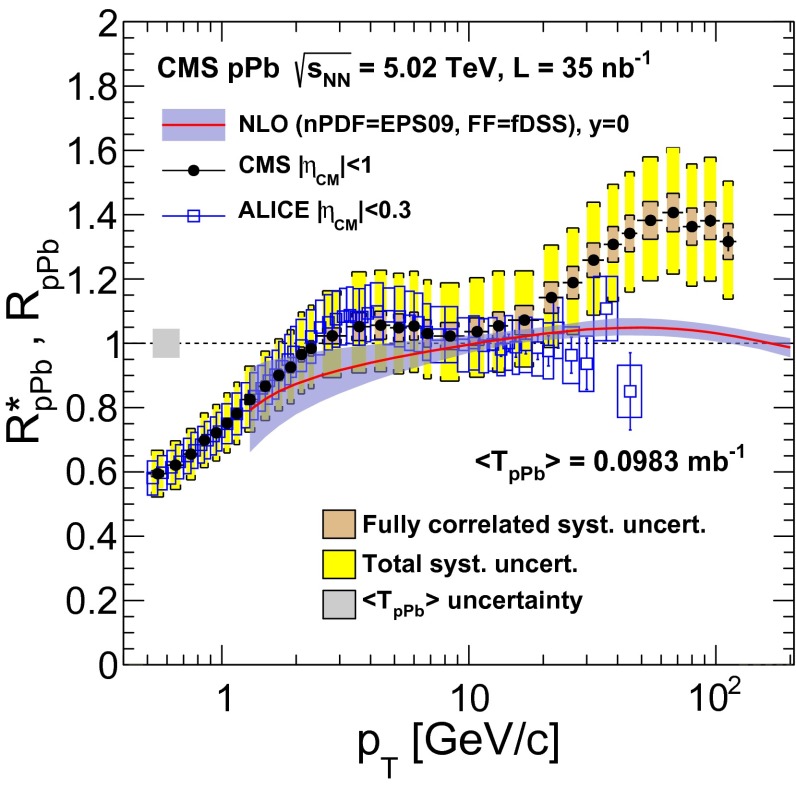
Charged-particle nuclear modification factors measured by CMS in $|\eta _\textsc {cm} |<1$ (*filled circles*), and by ALICE in $|\eta _\textsc {cm} |<0.3$ (*open squares*), are compared to the NLO pQCD prediction of Ref. [53]. The theoretical uncertainty is based on the EPS09 error sets. For the CMS measurement, the *shaded band* at unity and $p_{\mathrm {T}} \approx 0.6$ represents the uncertainty in the Glauber calculation of $\langle \mathrm {T}_\mathrm {pPb} \rangle$, the *smaller uncertainty band* around the data points shows the fully correlated uncertainties and the total systematic uncertainty is shown by the *larger band* (see Table 1). For the ALICE measurement, the total systematic uncertainties, excluding the normalization uncertainty of 6 %, are shown with *open boxes*

In Fig. 5, the CMS measurement is compared to the result of an NLO pQCD calculation [53] for charged particles produced at mid-rapidity. The calculation uses the CTEQ10 [54] free-proton PDF, the EPS09 nPDF [4], and the fDSS fragmentation functions [55]. The observed rise of the nuclear modification factor up to $R^{*}_\mathrm {pPb} \approx 1.3$–1.4 at high $p_{\mathrm {T}}$ is stronger than expected theoretically. None of the available theoretical models [52] predict enhancements beyond $R_\mathrm {pPb} \approx$ 1.1 at high $p_{\mathrm {T}}$. In particular, although the $p_{\mathrm {T}}$ range corresponds to parton momentum fractions $0.02\lesssim x \lesssim 0.2$, which coincides with the region where parton antishadowing effects are expected [10], none of the nPDFs obtained from global fits to nuclear data predict enhancements beyond 10 % at the large virtualities ($Q^2 \sim p_{\mathrm {T}} ^2 \sim 500\text {--}10{,}000{\,\text {GeV/}c^\text {2}}$) of relevance here.

An estimate of the significance of this observed rise above unity for $40 < p_{\mathrm {T}} < 120{\,\text {GeV/}c}$ is determined by interpreting all uncertainties as following a multivariate normal distribution where the components are the six $p_{\mathrm {T}}$ bins in the kinematic region of interest. The variance of each component is given by the sum of the statistical and systematic uncertainties in quadrature. For the case of the asymmetric particle species uncertainty, the smaller negative value is used as the data are uniformly larger than the expected values of the hypothesis to be tested. Given that the uncertainties of the reference spectrum are derived from applying different interpolation procedures and propagating the uncertainties from previous measurements from multiple experiments, it is not possible to unambiguously determine how all systematic uncertainties are correlated between measurements in each $p_{\mathrm {T}}$ bin. Therefore, a pair of estimates of the possible significance is given. In one case, only the systematic uncertainties from the relative normalization of the spectra, track selection, trigger efficiency, nuclear thickness function, and NLO pQCD calculation are treated as fully correlated, while others are treated as uncorrelated. In the other case, all systematic uncertainties are treated as fully correlated. Both the hypothesis that $R^{*}_\mathrm {pPb}$ is unity and the hypothesis that $R^{*}_\mathrm {pPb}$ is given by the NLO pQCD calculation are tested. For the case in which some uncertainties are treated as uncorrelated, a log-likelihood ratio test is performed using an alternative hypothesis that $R^{*}_\mathrm {pPb}$ is given by either unity or the NLO prediction, scaled by a constant, $p_{\mathrm {T}}$-independent, factor. The hypothesis that $R^{*}_\mathrm {pPb}$ is unity for $40 < p_{\mathrm {T}} < 120{\,\text {GeV/}c}$ is rejected with a $p$ value of 0.006 %, and the hypothesis that $R^{*}_\mathrm {pPb}$ is given by the NLO pQCD calculation for $40 < p_{\mathrm {T}} < 120{\,\text {GeV/}c}$ is rejected with a $p$ value of 0.2 %. For the case in which all uncertainties are fully correlated, the log-likelihood ratio test cannot be used, as the covariance matrix becomes nearly singular and the maximum likelihood estimation fails. Instead, a two-tailed univariate test is performed using the single measurement for $61 < p_{\mathrm {T}} < 74{\,\text {GeV/}c}$. From this test, the hypothesis that $R^{*}_\mathrm {pPb}$ is unity for $61 < p_{\mathrm {T}} < 74{\,\text {GeV/}c}$ is rejected with a $p$ value of 0.4 %, and the hypothesis that $R^{*}_\mathrm {pPb}$ is given by the NLO pQCD calculation for $61 < p_{\mathrm {T}} < 74{\,\text {GeV/}c}$ is rejected with a $p$ value of 2 %.

Figure 5 also shows the measurement from the ALICE experiment [22], which is performed in a narrower pseudorapidity range than the CMS one, and uses a different method (NLO scaling) to obtain the pp reference spectrum based on ALICE pp data measured at $\sqrt{s} = 7$ $\,\text {TeV}$. The difference in the CMS and ALICE $R^{*}_\mathrm {pPb}$ results stems primarily from differences in the charged-hadron spectra measured in pp collisions at $\sqrt{s} = 7$ $\,\text {TeV}$  [38, 56].

**Fig. 6:**
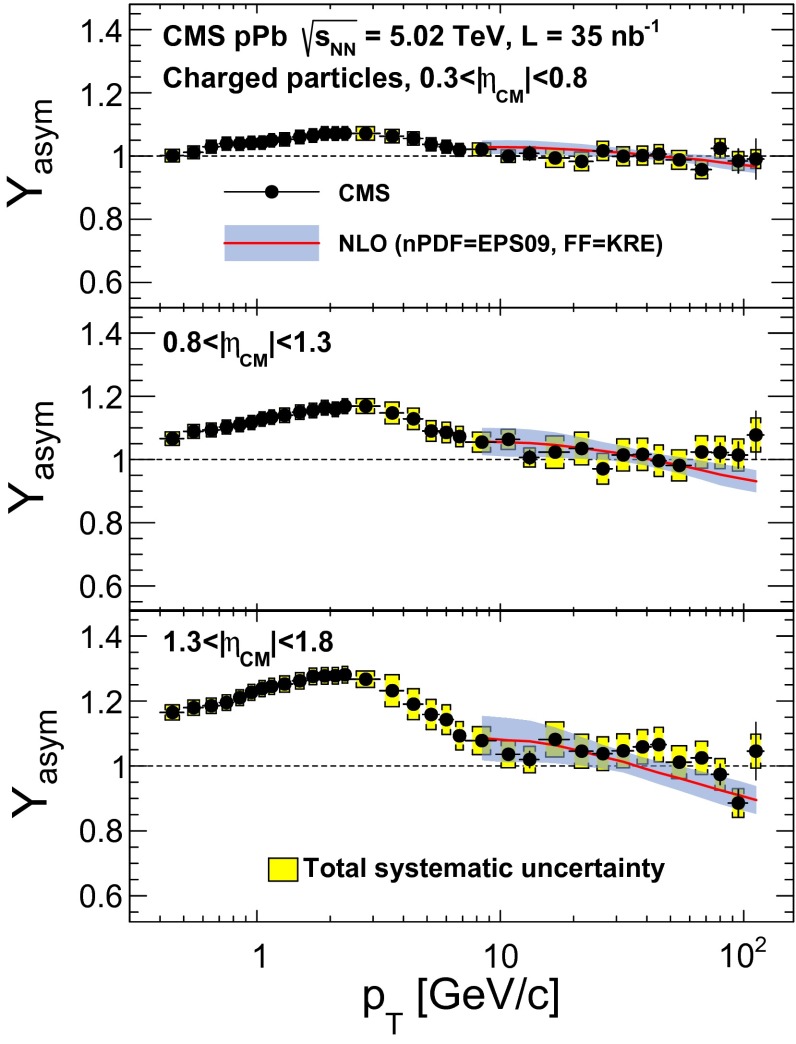
Charged-particle forward-backward yield asymmetry as a function of $p_{\mathrm {T}}$ for $0.3<|\eta _\textsc {cm} |<0.8$ (*top*), $0.8<|\eta _\textsc {cm} |<1.3$ (*middle*), and $1.3<|\eta _\textsc {cm} |<1.8$ (*bottom*). The asymmetry is computed as the charged-particle yields in the direction of the Pb beam divided by those of the proton beam. The *solid curves* are NLO pQCD theoretical calculations including nPDFs modifications [53]. The theoretical uncertainty is based on the EPS09 error sets

Figure 6 shows the forward-backward yield asymmetry, $Y_\text {asym}$ (Eq. 2), as a function of $p_{\mathrm {T}}$ for $0.3<|\eta _\textsc {cm} |<0.8$, $0.8<|\eta _\textsc {cm} |<1.3$, and $1.3<|\eta _\textsc {cm} |<1.8$. In all three $\eta$ ranges, the value of $Y_\text {asym}$ rises from $p_{\mathrm {T}} \approx 0.4$ to about 3${\,\text {GeV/}c}$, then falls to unity at a $p_{\mathrm {T}}$ of 10${\,\text {GeV/}c}$, and remains constant at unity up to the highest $p_{\mathrm {T}}$ values. At the lowest $p_{\mathrm {T}}$ value, $Y_\text {asym}$ is consistent with unity for $0.3<|\eta _\textsc {cm} |<0.8$, but is above unity in the larger pseudorapidity regions. For $p_{\mathrm {T}} < 10 {\,\text {GeV/}c}$, the $Y_\text {asym}$ is larger than unity as has been predicted by models including nuclear shadowing [52]. A theoretical NLO pQCD computation of $Y_\text {asym}$ at high $p_{\mathrm {T}}$  [53], using CTEQ6 [57] free-proton PDFs, EPS09 nPDFs [4], and Kretzer parton-to-hadron fragmentation functions [58], is also shown in Fig. 6. The theoretical predictions are consistent with these data.

To determine if the $R^{*}_\mathrm {pPb}$ and $Y_\text {asym}$ results can be consistently interpreted in terms of nPDF modifications, an MC study using the pythia (Z2 tune) event generator was performed to correlate each high-$p_{\mathrm {T}}$ hadron to the fractional momentum, $x$, of the initial-state parton from the Pb nucleus that participated in the hard-scattering process producing the final hadron. In all pseudorapidity intervals studied here, most of the hadrons with $p_{\mathrm {T}} \gtrsim 20$ ${\,\text {GeV/}c}$, i.e., in the range where the $R^{*}_\mathrm {pPb}$ exceeds unity in Fig. 4, come from the $x$ region that is associated with antishadowing in the nPDF distributions. Although the mean of the $x$ distribution increases with $\eta _\textsc {cm}$, for hadrons with $p_{\mathrm {T}}$ above 20${\,\text {GeV/}c}$ it remains in the range $0.02\lesssim x \lesssim 0.2$. Thus, similar antishadowing effects are expected in the positive and negative $\eta _\textsc {cm}$ regions resulting in a $Y_\text {asym}$ close to unity. At low $p_{\mathrm {T}}$, corresponding to $x \lesssim 0.02$, a larger hadron yield is observed in the direction of the Pb beam. This is qualitatively consistent with expectations of gluon shadowing [52].

An enhancement in $R^{*}_\mathrm {pPb}$ at high $p_{\mathrm {T}}$ can possibly arise if the quark-jet fraction is larger in pPb than in pp collisions. Since the charged-particle products of quark fragmentation more often have higher relative $p_{\mathrm {T}}$ than those produced by gluon fragmentation, that could lead to an enhancement in the charged-particle production at high $p_{\mathrm {T}}$ beyond NLO expectations, without a corresponding increase in the jet $R_\mathrm {pPb}$ [25, 26]. We note that the gluon-to-hadron fragmentation functions are not well constrained in pp collisions at LHC energies [27], although such uncertainties should largely cancel in ratios of cross sections.

## Summary

Charged-particle spectra have been measured in pPb collisions at $\sqrt{s_{_\mathrm {NN}}} =5.02$ $\,\text {TeV}$ in the transverse momentum range of $0.4 < p_{\mathrm {T}} < 120$ ${\,\text {GeV/}c}$ for pseudorapidities up to $|\eta _\textsc {cm} | = 1.8$. The forward-backward yield asymmetry has been measured as a function of $p_{\mathrm {T}}$ for three bins in $\eta _\textsc {cm}$. At $p_{\mathrm {T}} < 10$ ${\,\text {GeV/}c}$, the charged-particle production is enhanced in the direction of the Pb beam, in qualitative agreement with nuclear shadowing expectations. The nuclear modification factor at mid-rapidity, relative to a reference spectrum interpolated from pp measurements at lower and higher collision energies, rises above unity at high $p_{\mathrm {T}}$ reaching an $R^{*}_\mathrm {pPb}$ value of 1.3–1.4 at $p_{\mathrm {T}} \gtrsim 40$ ${\,\text {GeV/}c}$. The observed enhancement is larger than expected from NLO pQCD predictions that include antishadowing effects in the nuclear PDFs in this kinematic range. Future direct measurement of the spectra of jets and charged particles in pp collisions at a center-of-mass energy of 5.02$\,\text {TeV}$ is necessary to better constrain the fragmentation functions and also to reduce the dominant systematic uncertainties in the charged-particle nuclear modification factor.
